# Global Transcriptomic Analysis of Targeted Silencing of Two Paralogous ACC Oxidase Genes in Banana

**DOI:** 10.3390/ijms17101632

**Published:** 2016-09-26

**Authors:** Yan Xia, Chi Kuan, Chien-Hsiang Chiu, Xiao-Jing Chen, Yi-Yin Do, Pung-Ling Huang

**Affiliations:** 1 College of Horticulture, Fujian Agriculture and Forestry University, Fuzhou 350002, China; xyfafu@163.com (Y.X.); 13763807691@163.com (X.-J.C.); 2 Department of Horticulture and Landscape Architecture, National Taiwan University, Taipei 10617, Taiwan; b02608009@ntu.edu.tw (C.K.); r01628127@ntu.edu.tw (C.-H.C.); 3 Graduate Institute of Biotechnology, Chinese Culture University, Taipei 11114, Taiwan

**Keywords:** fruit ripening, ethylene production, genetic transformation, RNA interference

## Abstract

Among 18 1-aminocyclopropane-1-carboxylic acid (ACC) oxidase homologous genes existing in the banana genome there are two genes, *Mh-ACO1* and *Mh-ACO2*, that participate in banana fruit ripening. To better understand the physiological functions of *Mh-ACO1* and *Mh-ACO2*, two hairpin-type siRNA expression vectors targeting both the *Mh-ACO1* and *Mh-ACO2* were constructed and incorporated into the banana genome by *Agrobacterium*-mediated transformation. The generation of *Mh-ACO1* and *Mh-ACO2* RNAi transgenic banana plants was confirmed by Southern blot analysis. To gain insights into the functional diversity and complexity between *Mh-ACO1* and *Mh-ACO2*, transcriptome sequencing of banana fruits using the Illumina next-generation sequencer was performed. A total of 32,093,976 reads, assembled into 88,031 unigenes for 123,617 transcripts were obtained. Significantly enriched Gene Oncology (GO) terms and the number of differentially expressed genes (DEGs) with GO annotation were ‘catalytic activity’ (1327, 56.4%), ‘heme binding’ (65, 2.76%), ‘tetrapyrrole binding’ (66, 2.81%), and ‘oxidoreductase activity’ (287, 12.21%). Real-time RT-PCR was further performed with mRNAs from both peel and pulp of banana fruits in *Mh-ACO1* and *Mh-ACO2* RNAi transgenic plants. The results showed that expression levels of genes related to ethylene signaling in ripening banana fruits were strongly influenced by the expression of genes associated with ethylene biosynthesis.

## 1. Introduction

Banana is a typical climacteric fruit of high economic importance that originated in Southeast Asia [1,2]. Ethylene production of climacteric fruits has been proposed to regulate many developmental events and abiotic stress responses of plants. As one of the physiological processes, fruit ripening is most sensitive to ethylene [3]. The quality and storage life of banana are affected by ethylene. In general, the climacteric ripening process involves two systems: system I is responsible for a ripening-related increase in respiration, and system II operates an autocatalytic biosynthesis and a burst of ethylene production concomitantly with physicochemical and biochemical changes [4,5]. Ethylene biosynthesis begins from *S*-adenosylmethionine (SAM); SAM is synthesized from methionine and ATP, then 1-aminocyclopropane-1-carboxylic acid (ACC) synthase (ACS) and ACC oxidase (ACO) catalyze the two key steps of the ethylene biosynthesis pathway [6,7]. First, the rate-limiting step catalyzed by ACS involves the cyclization of SAM to ACC, and 5′-methylthioadenosine (MTA) is produced by ACS to utilize the synthesis of new methionine by the activated methyl cycle. This salvage pathway preserves the methyl group for another round of ethylene production. Finally, ACO catalyzes the oxygen-dependent conversion of ACC to ethylene [3,8]. ACC synthase is encoded by medium-sized gene families and the members are spatially and temporally regulated by various developmental, environmental, and hormonal signals [9]. The first ACC synthase, cDNA, was isolated from zucchini using an experimental approach [10]. Since the initial reports, ACC synthase, or genomic sequences, were isolated from numerous climacteric fruits such as apple [11], papaya [12], kiwifruit [13], sugarcane [14], peach [15], banana [16], tomato [17], and pear [18].

In climacteric fruit, a large amount of ethylene usually releases at the beginning of the climacteric respiratory period, exogenous ethylene induction, and endogenous ethylene production. In *Arabidopsis thaliana*, ethylene response 1 (ETR1), ethylene response 2 (ETR2), ethylene response sensor 1 (ERS1), ethylene response sensor 2 (ERS2), and ethylene-insensitive 4 (EIN4) are components of the five-membered family of ethylene receptors [19,20,21]. In the ethylene-signaling pathway, the receptors act like negative regulators through activation of the Serine/Threonine kinase constitutive triple response 1 (CTR1) [22]. As a key mediator of ethylene signal transduction, CTR1 acts as downstream receptor in transmitting the ethylene signal, and one of the substrates for CTR1 is ethylene insensitive 2 (EIN2). The EIN2 protein, an endoplasmic reticulum-bound protein, shows similarity to natural resistance associated macrophage protein (NRAMP) metal-ion transporters that are maintained in an inactive state when phosphorylated by CTR1. This phosphorylation prevents the EIN2 C-terminal domain from migrating into the nucleus [23,24]. In the absence of the ethylene precursor ACC, EIN2 was relocated to the endoplasmic reticulum (ER) and accumulated in the nucleus upon exposure to ethylene through nuclear localization signal (NLS). Downstream of EIN2, ethylene signaling is facilitated by nuclear protein EIN3/EIN3-like (EIL) transcriptional responses to activate ethylene response factor (ERF) genes by binding to specific *cis*-elements in promoter regions [23,25].

As a typical climacteric fruit, ethylene production in banana fruit is regulated by transcription of ACC synthase until climacteric rise and by reduction of ACC oxidase activity [5,16]. Banana *MA-ACS1* mRNA accumulated at the onset of the climacteric period, and *MA-ACO1* gene encoding banana ACC oxidase was detectable at the preclimacteric stage, then elevated throughout the final stage in the climacteric and postclimacteric phase [26,27]. Similarly, two ACC oxidase genes, *Mh-ACO1* (GenBank accession number AF030411) and *Mh-ACO2* (GenBank accession number U86045, *MA-ACO1* paralog), were found to be involved in banana fruit ripening [28]. Gene expression of *Mh-ACO1* was greatly induced at an early stage of fruit ripening in pulp and showed maximal expression at stage 6 [28]. The expression of *Mh-ACO2* gene in banana begins after the onset of ripening (stage 2) and continues into later stages of the ripening process [16]. Moreover, the abundance of *Mh-ACO2* mRNA transcripts in pulp had increased dramatically by stage 6 [28]. Both *Mh-ACO1* and *Mh-ACO2* genes, differentially expressed in fruits and other organs of banana, contribute to the increase of ethylene production in banana fruits [16,28]. Compared with other ACC oxidases, *Mh-ACO1* lacks the putative leucine zipper motif and shares less than 50% homology with known ACC oxidase in other climacteric fruit. Both *Mh-ACO1* and *Mh-ACO2* contributed to increased ethylene production and modulate enzyme activity in this ripening process. In this research, RNA interference (RNAi) targeting one of the two ACC oxidase genes was used to further investigate the differential functions of *Mh-ACO1* and *Mh-ACO2* during fruit ripening. Gene expression profiles have been conducted during banana fruit ripening by taking advantage of the next-generation high-throughput sequencing to sequence the transcripts of *Mh-ACO1* and *Mh-ACO2* RNAi transgenic banana fruits. Furthermore, the analysis presented in this study identified many novel biological functions regulating fruit ripening development for the first time and highlighted more comprehensively important biological processes associated with fruit ripening in banana.

## 2. Results

### 2.1. Chromosomal Distribution of the 1-Aminocyclopropane-1-carboxylic acid (ACC) Oxidase Genes on the Banana Genome

**Figure 1:**
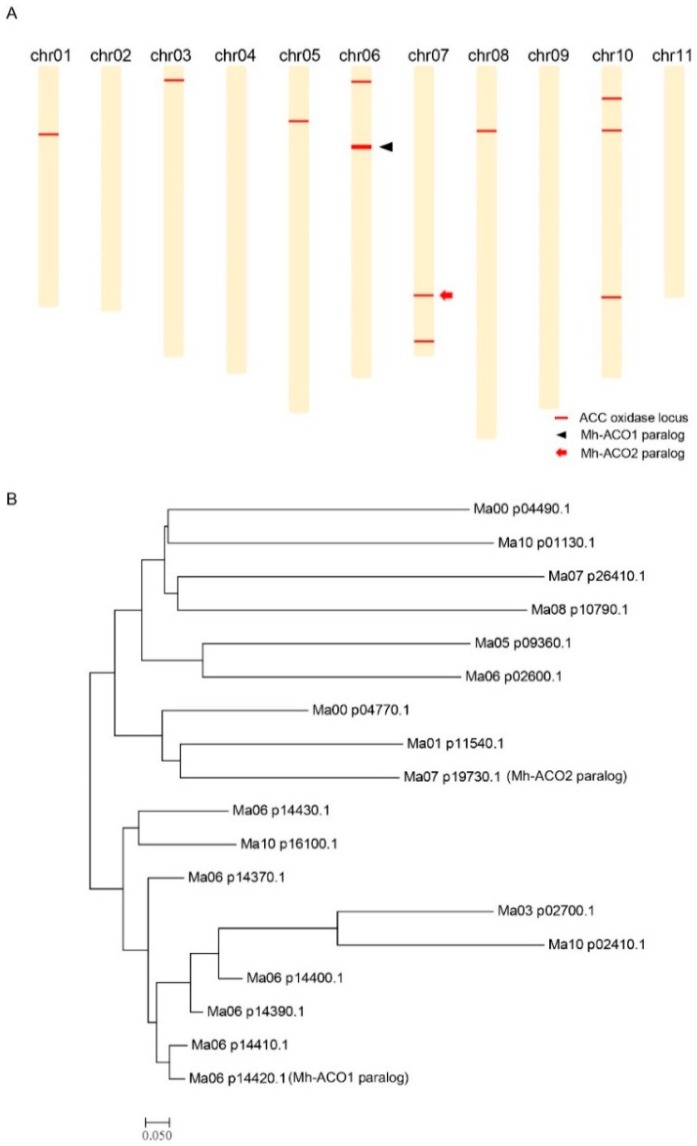
Chromosomal distribution and amino acid sequence analysis of the 1-aminocyclopropane-1-carboxylic acid (ACC) oxidase homologous genes on the *Musa acuminata* genome. (**A**) Chromosomal distribution of the 18 ACC oxidase homologous genes on the *Musa acuminata* genome. The black arrow indicates the *Mh-ACO1* paralog where six ACC oxidase homologous genes are clustered. The red arrow indicates the *Mh-ACO2* paralog. Two unidentified loci, namely Ma00_p04990.1 and Ma00_p04770.1, are not shown; (**B**) Amino acid sequence-based dendrogram among 18 ACC oxidase homologs. The Mh-ACO1 paralog and Mh-ACO2 paralog are defined using the Blastx tool in the Banana Genome Hub [31] The list of each entry is shown as Table S1.

In the banana genome, there are 18 ACC oxidase homologous genes on the *Musa acuminata* genome, based on the search results shown in Figure 1A in the Banana Genome Hub [29,30] using the Blastx tool [31]. The amino acid sequence-based dendrogram among these 18 ACC oxidase homologs is shown in Figure 1B. The distributions of these genes are located as follows: there exists only one gene distributed on chromosomes 1, 3, 5, and 8, respectively. Seven genes are located on chromosome 6. Two genes are located on chromosome 7. Three genes are located on chromosome 10. Additionally, there are two unidentified loci, namely Ma00_p04990.1 and Ma00_p04770.1. Detailed information containing gene ID, chromosomal distribution, and gene positions of these 18 ACC oxidase homologs on the *Musa acuminata* genome are shown in Table S1.

### 2.2. Generation of Two RNA Interference Transgenic Banana Plants by Expressing siRNA for Mh-ACO1 and Mh-ACO2

**Figure 2:**
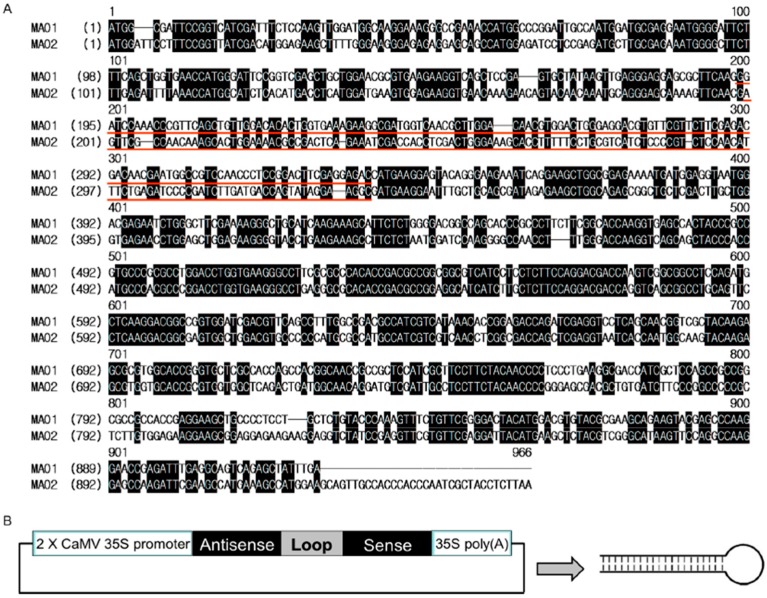
RNA interference by expressing siRNA for *Mh-ACO1* and *Mh-ACO2* genes. (**A**) DNA sequences from *Mh-ACO1* and *Mh-ACO2* genes used for siRNA expression. Two siRNA-expressing plasmid vectors were constructed, in which siRNA-encoding regions were inserted downstream of the 2× *CaMV 35S* promoter. The regions used as antisense and sense fragments for the siRNA synthesis are indicated by red underlining. Nucleotides identical in both cDNAs are indicated as black boxes. The first intron of *Mh-ACO1* was used as spacer and shown as loop; (**B**) Diagrammatic presentation of stem loop-type siRNA driven by an enhanced double *CaMV 35S* promoter.

**Figure 3:**
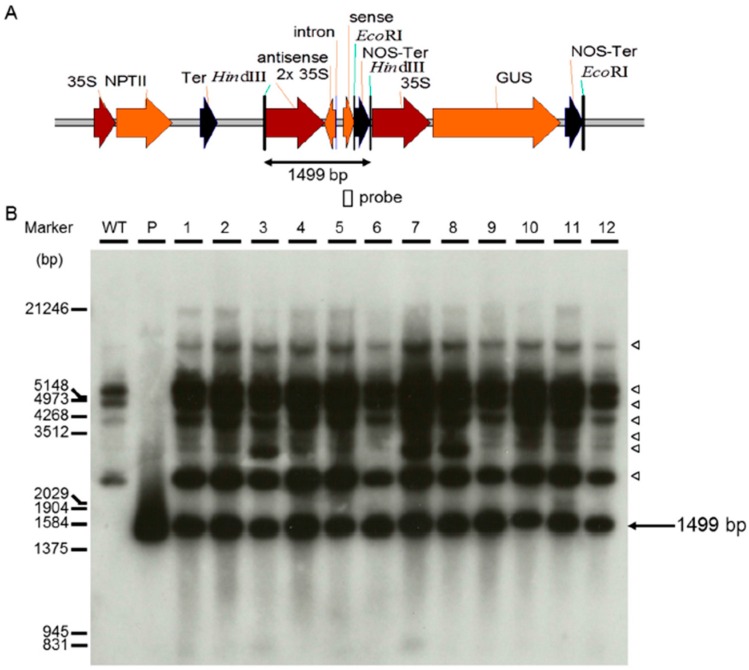
Southern blot analysis of transformed banana *Mh-ACO1 RNAi* plants. (**A**) Schematic diagram of the T-DNA region in the plasmid construct pBI121-1AnS used for banana transformation. The positions of hybridization probe and the expected hybridization fragment are also shown. 35S, *Cauliflower Mosaic Virus* (*CaMV*) *35S* promoter; 2× 35S, an enhanced double *CaMV 35S* promoter; antisense, *Mh-ACO1*-specific antisense fragment; intron, *Mh-ACO1* intron fragment*;* sense, *Mh-ACO1*-specific sense fragment; NPTII, neomycin phosphotransferase II gene; GUS, the β-glucuronidase gene; Ter, *CaMV 35S* terminator; NOS-Ter, nopaline synthase terminator; (**B**) Autoradiogram for Southern analysis of 11 independent transgenic lines. Genomic DNA was digested with *Hin*dIII, and hybridized with ^32^P-labelled *Mh-ACO1* specific fragment (149 bp) as probe. Hybridized endogenous gene fragments of ACC oxidase are indicated by hollow triangles. P—plasmid DNA of pBI121-1AnS as positive control. WT—untransformed plant.

**Figure 4:**
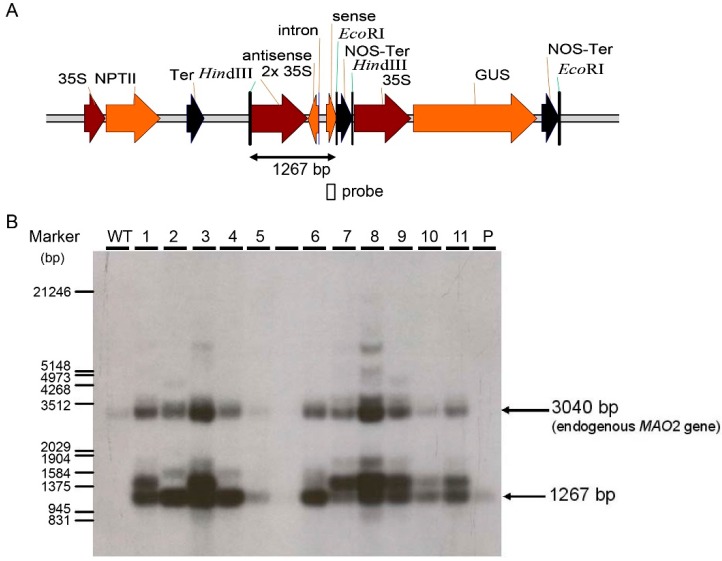
Southern analysis of putative transformed banana *Mh-ACO2 RNAi* plants. (**A**) Partial diagram of plasmid transformed. 35S, *CaMV 35S* promoter; 2× 35S, an enhanced double *CaMV 35S* promoter; antisense, *Mh-ACO1*-specific antisense fragment; intron, *Mh-ACO1* intron fragment*;* sense, *Mh-ACO1*-specific sense fragment; NPTII, neomycin phosphotransferase II gene; GUS, the β-glucuronidase gene; Ter, *CaMV 35S* terminator; NOS-Ter, nopaline synthase terminator; (**B**) Southern analysis of 11 independent transgenic lines using *Mh-ACO2* cDNA as probe. P—transformed plasmid as positive control; WT—untransformed control plant. A total of 20 mg of genomic DNA was digested with *Eco*RI and *Hin*dIII and subjected to a 0.7% agarose gel.

Among 18 ACC oxidase homologous genes on the banana genome there are two ACC oxidase genes, *Mh-ACO1* [28] and *Mh-ACO2* [16], that play pivotal roles in the fruit growth and development of banana ripening. Both *Mh-ACO1* and *Mh-ACO2* contribute to increased ethylene production in fruits, and are differentially expressed in fruits and other organs in banana. To better understand the physiological functions of *Mh-ACO1* and *Mh-ACO2* involved in regulating the ripening process of banana fruits by using RNA interference (RNAi) technology, two siRNA-expressing plasmid vectors were constructed, in which siRNA-encoding regions were inserted downstream of the 2× *CaMV 35S* promoter. The regions from *Mh-ACO1* and *Mh-ACO2* used as antisense and sense fragments for the siRNA synthesis are indicated in Figure 2A. The first intron of *Mh-ACO1* [28] was used as a spacer for the siRNA expression as shown in Figure 2B. Using *Agrobacterium*-mediated transformation system with banana embryogenic cells, two populations of RNAi transgenic banana plants, *Mh-ACO1* (As1) and *Mh-ACO2* (As2) RNAi, were generated for the expression of siRNA corresponding to the targeted regions from *Mh-ACO1* and *Mh-ACO2*, respectively. All transgenic RNAi banana plants were screened by GUS histochemical staining [32], analyzed by PCR and proved by Southern blot analysis. As shown in Figure 3 and Figure 4, two representative subsets of *Mh-ACO1* and *Mh-ACO2* RNAi plant populations, respectively, demonstrate the integration of transgenes in the banana genome. In a relevant study we have measured the ethylene emission in the fruits from untransformed (WT) and two RNAi transgenic banana lines. It clearly showed apparent gene-silencing effects of fruit ripening for both *Mh-ACO1* and *Mh-ACO2* silenced transgenic banana plants. The preliminary data regarding the silencing effects of fruit ripening for both *Mh-ACO1* and *Mh-ACO2*-silenced transgenic banana plants is shown in Table S2.

### 2.3. Isolation of Total RNA from Mh-ACO1 and Mh-ACO2 RNAi Plants for RNA-Sequence Analysis

In order to achieve a banana transcriptome, total RNA was extracted from untransformed (WT), *Mh-ACO1* silenced transgenic, and *Mh-ACO2* silenced transgenic plants, respectively. The RNA samples, of which the quality was determined by agarose gel electrophoresis and OD_260_/OD_280_ ratio (2.0 ± 0.10), were found to be suitable for cDNA synthesis. The normalized cDNA significantly reduces the generation of repetitive sequences and increases the rate recovery of unique transcripts. After quality control experiments, standardized cDNA was used to construct the cDNA library, and then the library was sequenced by HiSeq^TM^ Illumina 2500 (Illumina, Inc., San Diego, CA, USA).

### 2.4. Assembly of Reads and Transcriptome Characterization of Mh-ACO1 and Mh-ACO2 RNAi Plants by High-Throughput RNA Sequencing

The original raw data from Illumina HiSeq^TM^ 2500 were transformed to sequenced reads by base calling. The raw and processed data from this study have been submitted to the NCBI Gene Expression Omnibus [33] under the series accession numbers SRR3732000, SRR3732001, and SRR3732002. After filtering and removing reads containing adapters or reads of low quality, the total clean reads of WT, As1 and As2, 2,068,587 (96.42%), 16,046,988 (97.51%), and 16,279,746 (97.32%) were obtained, respectively. After clustering and assembly, a total of 123,617 high quality transcripts with a total size of 111,501,484 bp and 88,031 unigenes were obtained. Length of these transcripts ranged between 201 and 16,049 bases, most of these transcripts and unigenes are in the 200–500 bp length range. Therefore, this transcriptome dataset provides a valuable and comprehensive resource for further analysis on targeted silencing of two paralogous ACC oxidase genes in banana.

For annotation, all the unigenes were aligned by Blastx to the NR (NCBI non-redundant protein database) [34], NT (NCBI nucleotide database) [34], KO (KEGG Orthology) [35], Swiss-Prot [36], PFAM (Protein Families) [37], GO [38], and eukaryotic Orthologous Groups (KOG) [39] databases. Using a cut-off *E*-value of 10^−5^, 37,679 (42.8%), 42,325 (48.07%), 12,163 (13.81%), 24,931 (28.32%), 24,104 (27.38%), 24,411 (27.73%), and (13.66%) unigenes were successfully annotated in the seven databases, respectively. While 6300 (7.15%) unigenes were successfully annotated in all the seven databases, 46,904 (53.28%) were successfully annotated in at least one database.

Those unigenes with no-match hits in the protein database might be due to their too-short sequence length. In terms of genetic similarity, approximately 91.1% of the sequences have more than 80% similarity with known genes, and 80.3% of the sequences have more than 95% similarity with known genes. As for annotation of species distribution, 91.3% had top matches (first hit) with sequences from banana (*Musa acuminata*), 1.5% from date (*Phoenix dactylifera*), 0.5% and 0.4% from rice (*Oryza sativa*) and grape (*Vitis vinifera*), respectively.

### 2.5. Transcriptome Characterization of Mh-ACO1 and Mh-ACO2 RNAi Plants by High-Throughput RNA Sequencing

#### 2.5.1. Gene Oncology (GO) Assignments

Gene ontology (GO) enrichment analysis was performed to classify the gene function of unigenes. According to the NR database annotation, a total of 24,411 (27.73%) unigenes can be categorized into 45 functional groups consisting of three domains: ‘biological process,’ ‘cellular component,’ and ‘molecular function,’ as shown in Figure S1.

#### 2.5.2. Eukaryotic Orthologous Groups (KOG) Annotation

For all annotated sequences for the genes involved in eukaryotic homologous protein clusters (KOG, euKaryotic Orthologous Groups) assignment, 12,031 unigenes have a KOG classification as shown in Figure S2.

#### 2.5.3. Kyoto Encyclopedia of Genes and Genomes (KEGG) Pathway Mapping

KEGG (Kyoto Encyclopedia of Genes and Genomes) database was used as an alternative approach to categorize gene function with emphasis on biochemical pathways. In total, 12,163 unigenes were assigned to 32 KEGG pathways, and the genes involved in KEGG metabolic pathways are divided into five branches. Detailed information is shown in Figure S3. These annotations provided a valuable clue for predicting potential genes and their functions at a whole-transcriptome level.

#### 2.5.4. Gene Expression Difference Analysis

A total of 6777 differentially expressed genes (DEGs) were detected with 3426 up-regulated and 3351 down-regulated genes. There were 3050 DEGs detected with 1590 up-regulated and 1460 down-regulated genes in the comparison of As1-vs-WT samples; there were 500 up-regulated and 443 down-regulated genes found in the comparison of As1-vs-As2 samples; a total of 2784 DEGs were detected with 1336 up-regulated and 1448 down-regulated genes in the comparison of As2-vs-WT samples (Figure S4).

#### 2.5.5. GO Enrichment Analysis of Differentially Expressed Genes (DEGs)

Differentially expressed genes annotation (fold change ≥2 and ≤0.5) in As1-vs-WT, As2-vs-WT, and As1-vs-As2 were performed to categorize the biological function of DEGs. All the differentially expressed genes were mapped to terms in the GO database. A total of 734 annotated genes were obtained in As1-vs-As2, with 411 up-regulated and 323 down-regulated genes; there were 2351 DEGs detected with 1246 up-regulated and 1105 down-regulated genes in As1-vs-WT, whereas 2142 DEGs were detected with 1026 up-regulated and 1116 down-regulated genes in As2-vs-WT.

**Figure 5:**
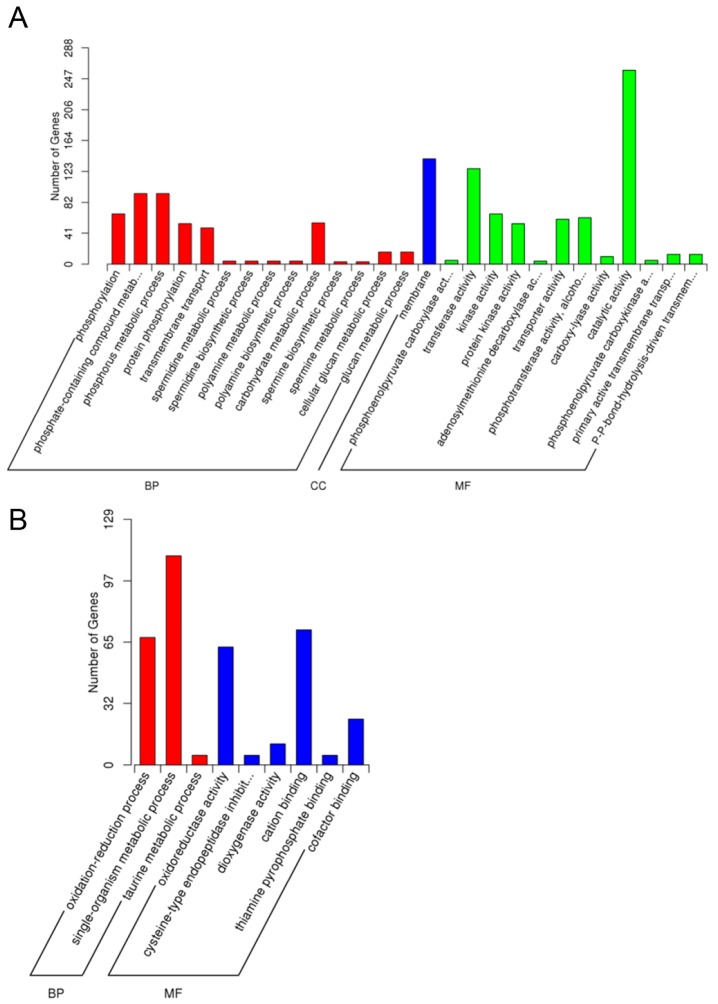

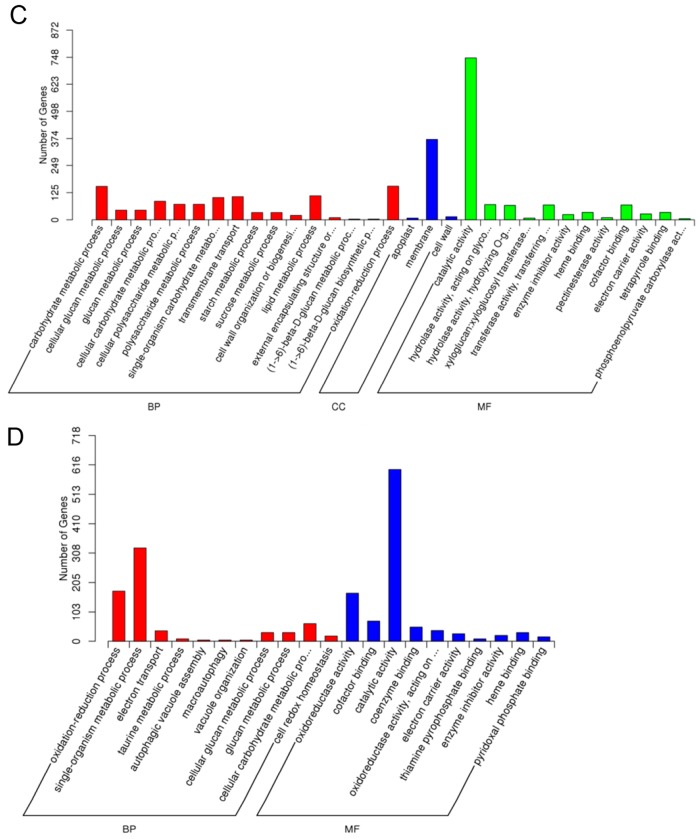
Gene Oncology (GO) enrichment analysis of differentially expressed genes (DEGs). (**A**) The GO enrichment result (As1-vs-As2) of up-regulated DEGs; (**B**) The GO enrichment result (As1-vs-As2) of down-regulated DEGs; (**C**) The GO enrichment result (As1-vs-WT) of up-regulated DEGs; (**D**) The GO enrichment result (As2-vs-WT) of up-regulated DEGs. GO with corrected *p*-value < 0.05 are significantly enriched in DEGs. The GO enrichment analysis results for As1-vs-WT and As2-vs-WT of down-regulated DEGs have shown no significance. BP—Biological process. MF—Molecular function. CC—Cellular component.

The GO enrichment results of all DEGs are significantly enriched in ‘molecular function’, ‘catalytic activity (GO:0003824)’, ‘heme binding (GO:0020037)’, ‘tetrapyrrole binding (GO:0046906)’, and ‘oxidoreductase activity (GO:0016491)’ comprised the largest proportion. A summary with the number and percentage of unigenes annotated in each GO slim term is shown. In As1-vs-As2, ‘catalytic activity’ (258, 62.77%), ‘transferase activity’ (258, 62.77%), and ‘membrane’ (127, 30.9%) represented the most common categories in up-regulated DEGs. ‘Single−organism metabolic process’ (110, 34.06%), ‘cation binding’ (71, 21.98%), ‘oxidation−reduction process’ (67, 20.74%), and ‘oxidoreductase activity’ (62, 19.2%) were among the most highly represented groups under down-regulated DEG annotations. Up-regulated in As1-vs-WT were mainly distributed in ‘catalytic activity’ (745, 59.79%) and ‘membrane’ (370, 29.7%). ‘Single−organism metabolic process’ (325, 31.68%), ‘catalytic activity’ (599, 58.38%), ‘oxidoreductase activity’ (168, 16.37%), and ‘oxidation−reduction process’ (175, 17.06%) were the highly represented groups under up-regulated DEGs in As2-vs-WT. Under the threshold of corrected *p*-value <0.05, the corrected *p*-values of down-regulated DEGs in As1-vs-WT and As2-vs-WT were 0.073506 and 0.069752, respectively, and the corrected *p*-values do not meet the requirement (Figure 5).

#### 2.5.6. KEGG Pathway Enrichment Analysis

**Figure 6:**
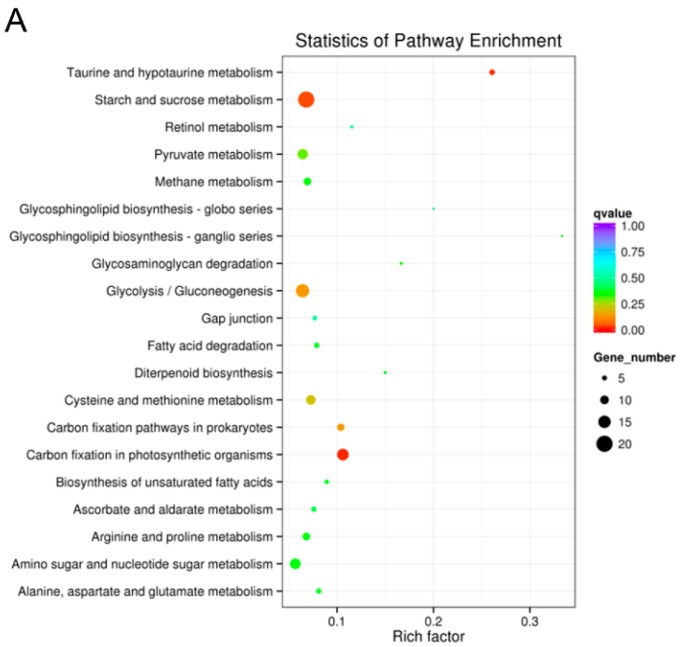

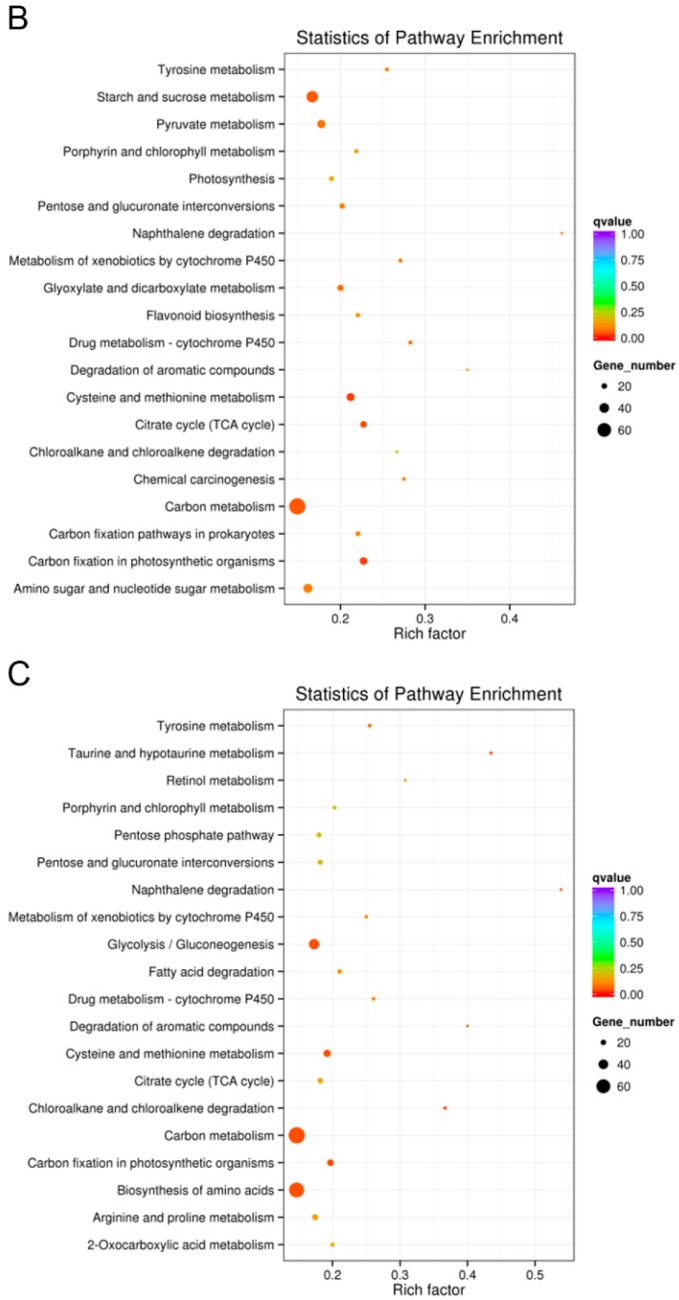
Kyoto Encyclopedia of Genes and Genomes (KEGG) enrichment scatter plot of DEGs. The *y*-axis represents the name of the pathway, and the *x*-axis represents the Rich factor. Dot size represents the number of different genes and the color indicates the q-value. (**A**) DEG enrichment analysis results (As1-vs-As2) in KEGG pathway; (**B**) DEG enrichment analysis results (As1-vs-WT) in KEGG pathway; (**C**) DEG enrichment analysis results (As2-vs-WT) in the KEGG pathway.

According to KEGG annotation, most of the DEGs were enriched in ‘carbon fixation in photosynthetic organisms’, ‘cysteine and methionine metabolism’, ‘citrate cycle (TCA cycle)’ and ‘starch and sucrose metabolism.’ According to the As1-vs-As2 DEGs enrichment analysis in KEGG pathway, 159 genes were annotated, and ‘starch and sucrose metabolism’ (20, 12.58%) was the most abundant, followed by ‘glycolysis/gluconeogenesis’ (16, 10.06%) and ‘carbon fixation in photosynthetic organisms’ (14, 8.81%). Up-regulated genes (98, 61.64%) were distributed in ‘amino sugar’ and ‘nucleotide sugar metabolism’ (11, 11.22%), and ‘starch and sucrose metabolism’ (11, 11.22%). Down-regulated DEGs (110, 69.2%) are mainly enriched in ‘glycolysis/gluconeogenesis’ (14, 12.73%), followed by ‘carbon metabolism’ (13, 11.82%) (Figure 6A). A total of 457 genes were annotated in As1-vs-WT, and most of the genes were enriched in ‘carbon metabolism’ (72, 15.75%), ‘starch and sucrose metabolism’ (49, 10.72%), and ‘amino sugar and nucleotide sugar metabolism’ (37, 8.10%). Among the up-regulated genes (347, 75.93%), ‘carbon metabolism’ (47, 13.26%), and ‘protein processing in endoplasmic reticulum’ (36, 10.37%) comprised the largest proportion. ‘Spliceosome’ (22, 10.23%) and ‘starch and sucrose metabolism’ (22, 10.23%) were among the most highly represented groups under down-regulated (215, 47.01%) DEGs (Figure 6B). Of 438 DEGs in As2-vs-WT, ‘carbon metabolism’ (71, 16.21%) and ‘biosynthesis of amino acids’ (65, 14.84%) account for a large fraction of the overall assignments, followed by ‘glycolysis/gluconeogenesis’ (43, 9.82%). ‘Carbon metabolism’ (49, 18.22%), and ‘biosynthesis of amino acids’ (42, 15.61%) were highly enriched in up-regulated genes (269, 61.42%), while ‘ribosome’ (28, 16.18%) and ‘starch and sucrose metabolism’ (21, 12.14%) were highly enriched in down-regulated genes (173, 39.50%) in As2-vs-WT (Figure 6C).

### 2.6. Expression of the Genes Involved in Ethylene Biosynthesis and Signal Transduction Pathway in Mh-ACO1 and Mh-ACO2 RNAi Transgenic Banana Fruits

**Figure 7:**
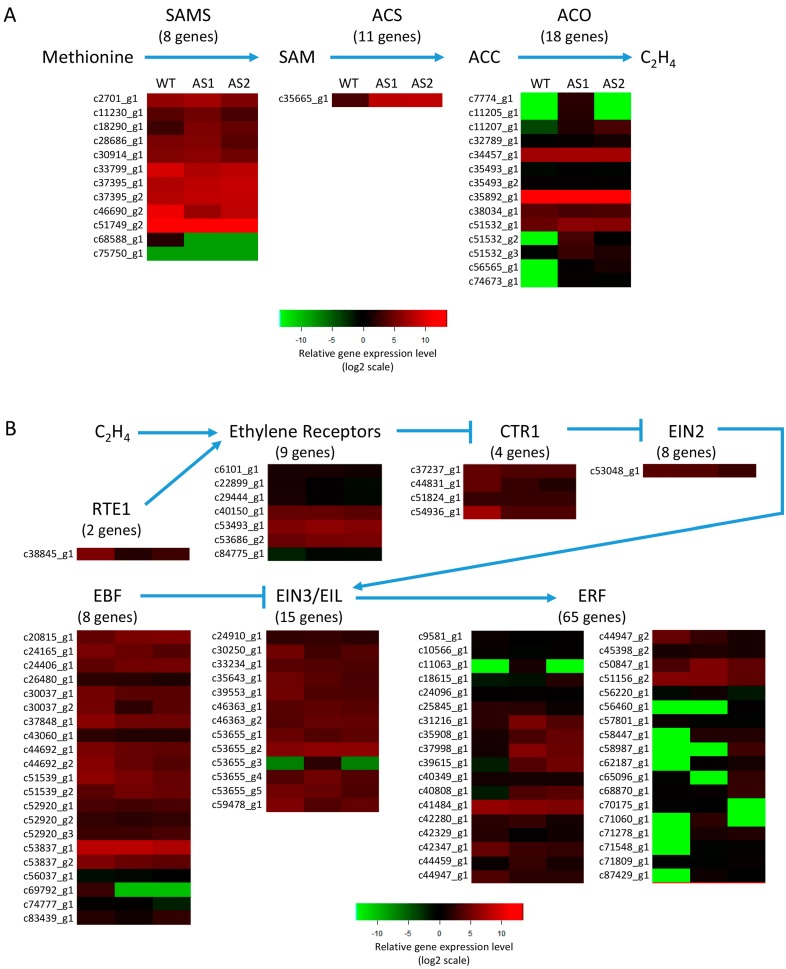
Expression of ethylene biosynthesis- and signaling-related genes in *Mh-ACO1 RNAi* (As1) and *Mh-ACO2 RNAi* (As2) banana plants. (**A**) Ethylene biosynthesis-related genes; (**B**) Ethylene signaling-related genes. Heatmaps indicate log2 fold change expression levels. Full data are given in Table S4. Of the 88,031 assembled contig IDs in WT, As1, and As2 unigene libraries, only those IDs that uniquely match to genes annotated in the Banana Genome Hub with pairwise identity of greater than 95% using BlastN are used to build the heat maps. The names of the selected IDs are indicated to the left of the histograms. The number of genes indicated below each respective gene is analyzed by a Blast search based on the Banana Genome Hub [31]. SAMS—*S*-adenosylmethionine (SAM) synthetase; ACS—1-aminocyclopropane-1-carboxylic acid (ACC) synthase; ACO, ACC oxidase; ETR1—ethylene response 1, CTR1—Constitutive triple response 1; EIN2—Ethylene insensitive 2; EIN3—Ethylene insensitive 3; EBF—EIN3-binding F-box protein; ERF—Ethylene responsive factor; RTE1—Reversion-to-ethylene-sensitivity 1.

As a typical climacteric fruit, the banana undergoes a ripening process characterized by a burst in ethylene production that triggers the initiation of ripening process. To gain a better understanding of the postharvest ripening of banana fruits at the molecular level, the genes related to ethylene biosynthesis and signal transduction were further selected for more comprehensive studies. Of the 88,031 assembled contigs in WT, As1 and As2 unigene libraries, only those IDs that uniquely match to genes annotated in the Banana Genome Hub with pairwise identity of greater than 95% using BlastN (as shown in Table S3) were used to analyze their gene expressions by heat map visualization based on fragments per kilobase of transcript per million mapped reads (FPKM) value (Table S4). The contig names which correspond to genes present in the Banana Genome Hub are listed in Figure 7. The majority of contig IDs were shown by high-throughput sequencing to be similar at stage 3 of fruit ripening in banana where the transcriptome analysis in this research was applied. However, contig c68588_g1, corresponding to SAM synthetase gene, contigs c7775_g1, c11205_g1, c51532_g1, c56565_g1, and c74673_g1, corresponding to ACC oxidase genes, contig c69792_g1, corresponding to EIN3-binding F-box protein gene, contig c53655_g1, corresponding to ethylene insensitive 3 gene, contigs c11063_g1, c56460_g1, c58447_g1, c58987_g1, c62187_g1, c65096_g1, c70175_g1, c71060_g1, c71278_g1, c71548_g1, and c87429_g1, corresponding to ethylene responsive factor genes, showed apparent change in levels of expression among WT, As1 and As2 banana samples. These differentially expressed genes would be very valuable for further study of their roles in fruit ripening.

**Figure 8:**
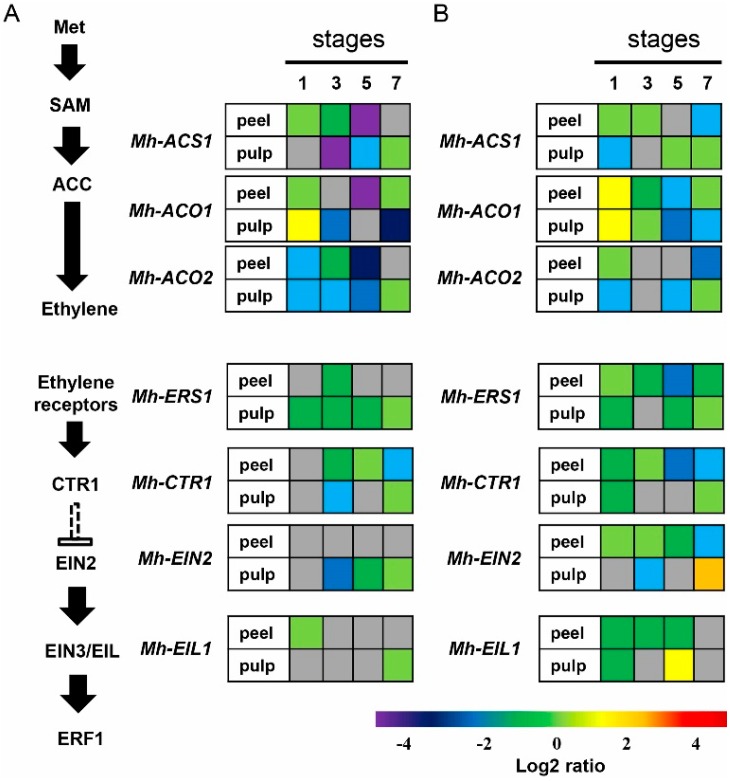
Expression profiles of the ethylene biosynthesis and signal transduction pathway genes and in *Mh-ACO1 RNAi* (**A**) and *Mh-ACO2 RNAi*; (**B**) transgenic banana fruits. Heatmap visualization of relative mRNA abundances was based on qRT-PCR data. Fruits were harvested at different stages that are indicated as four continuous blocks from left to right. Relative transcript abundance was normalized with the wild-type banana and transformed in log2. Gene expression levels are indicated with a rainbow color scale from purple (very weakly expressed) to red (very strongly expressed), whereas white boxes correspond to genes without detected expression, and grey boxes correspond to genes without differential expression between transgenic plant and wild-type. Data represent means of two biological replicates each determined in three technical replicates.

In order to validate the most significant expression profiles of the ethylene biosynthesis and signal pathway genes in *Mh-ACO1* and *Mh-ACO2*, for RNAi transgenic banana fruits at stages 1, 3, 5, and 7, real-time RT-PCR was performed with mRNAs from both peel and pulp of banana fruits. The changing patterns of these genes in the peel and pulp of ripening banana are shown in Table S5 and Figure 8. The results showed that the direction of expression change of all selected genes based on qPCR agreed with those detected by high-throughput sequencing. Both peel and pulp tissues showed the expression levels of a significant number of genes to be similar among banana samples of WT, As1, and As2. Some messages were clearly down-regulated, such as the patterns shown at stage 3 in pulp and those shown at stage 5 in peel for *Mh-ACS1*, *Mh-ACO1*, and *Mh-ACO2* in the *Mh-ACO1* RNAi transgenic banana fruits as shown in Figure 8A. Moreover, in the *Mh-ACO2* RNAi transgenic banana fruits, as shown in Figure 8B, the following observations were made: a significant reduction in gene expression levels of Mh-ACS1 (in peel at stage 3 and in pulp at stage 1); *Mh-ACO1* (in peel at stages 3 and 5, and in pulp at stages 5 and 7); *Mh-ACO2* (in peel at stage 7, and in pulp at stages 1 and 5); *Mh-ERS1* (in peel at stages 3, 5 and 7); and in pulp at stages 1 and 5); *Mh-CTR1* (in peel at stages 1, 5, and 7, and in pulp at stage 1); *Mh-EIN2* (in peel at stage 7, and in pulp at stage 3), and *Mh-EIL1* (in peel at stages 3, 5, and 7, and in pulp at stage 1). Additionally, expression of *Mh-EIN2* in pulp at stage 7 in *Mh-ACO2* RNAi transgenic banana fruits has been significantly up-regulated. These results suggest that both ethylene biosynthesis and signaling in ripening banana fruits may be controlled both in the peel and pulp tissues. A link between siRNA expressions for both *Mh-ACO1* and *Mh-ACO2* and gene expression levels in all investigated genes related to ethylene biosynthesis and signaling pathway is apparently an interesting issue to be elucidated.

## 3. Discussion

ACC oxidase plays a key role in ethylene biosynthesis for the conversion of ACC to ethylene [8]. However, 18 paralogs of ACC oxidase genes exist in the Banana Genome Hub using the Blastx tool. The contribution of each member of the ACC oxidase multigene family with respect to growth and development in banana life cycle remains to be elucidated. Banana fruits are consumed worldwide and are of great economic importance. Potentially, delayed ripening by ACC oxidase gene silencing can influence postharvest quality and storability of the fruit. This is evidenced by the fact that fruits from transgenic tomato plants whose ACC expression was suppressed by RNA interference were strongly reduced in ethylene production and delay in fruit ripening [40].

Considerable research has been carried out in the past to elucidate the molecular events of ACC oxidase gene involved in banana fruit ripening [41]. However, little information is available regarding the respective function of each member of ACC oxidase multigene family. In this research, a loss-of-function approach via RNA interference was used to explore the regulatory role of each ACC oxidase gene family. Banana fruits typically were harvested in the pre-climacteric stage (stage 1), while still green and hard, where very small quantities of ethylene were produced. Stage 3 is the transition stage just before banana fruits begin producing large amounts of ethylene. From this stage, banana fruits turned yellow and the entire fruit ripened onwards [42]. Therefore, the comparative transcriptome analysis of ripening fruits at stage 3 of WT, As1, and As2 was conducted to gain insight into the molecular mechanisms involved in fruit ripening. The results indicated that the down-regulation of many ethylene-related genes may be responsible for the decreased levels of expression observed in ethylene signaling genes. It is worthwhile to note that no remarkable change was observed between high-throughput transcriptomic data and the results obtained by real-time RT-PCR analysis. Several studies have demonstrated a close relationship between the expression levels of ethylene biosynthesis genes and those of ethylene signaling genes [41].

Transcriptomic comparison of enriched GO terms in DEGs for banana samples of As1 and As2 identifies four major GO terms with known or implicated functions (Figure 5). All these significantly expressed genes are involved in ‘catalytic activity,’ ‘heme binding,’ ‘tetrapyrrole binding,’ and ‘oxidoreductase activity.’ The results suggested a functional role of these genes involved in metabolic handling of oxygen species. Down-regulation of ACC oxidase genes whose translation products require dioxygen for activity affects oxygen metabolism [43]. 

Moreover, most of the up-regulated DEGs related to banana samples of As1 and As2 in KEGG enrichment analysis exhibited different expression patterns and levels relative to their wild-type (WT). These genes belong to the pathways of ‘carbon metabolism’ and ‘starch and sucrose metabolism,’ ‘biosynthesis of amino acids,’ and ‘glycolysis/gluconeogenesis’ (Figure 6), indicating a direct link between ethylene production and carbohydrate metabolism. Several enzymes, such as sucrose synthase and sucrose phosphate synthase, were known to be involved in starch-sucrose transformation, thereby resulting in the disappearance of the starch reserve during banana fruit ripening [44].

Degradation of RNA into short RNAs activates ribonucleases to target homologous mRNA and causes gene silencing [45]. Complete inhibition of a gene expression for either *Mh-ACO1* or *Mh-ACO2* was not observed in both *Mh-ACO1* and *Mh-ACO2* RNAi transgenic banana lines. The problems of using RNAi strategy to inactivate the gene function include: silencing efficiency, mRNA translatability, and post-translational regulation. However, the resulting physiological responses either in the reduction of ethylene synthesis or the increase of shelf-life in banana fruits in both *Mh-ACO1* and *Mh-ACO2* transgenic RNAi lines have been observed. There exists an apparent discrepancy between ethylene biosynthesis and the expression level of genes involved in ethylene biosynthesis. The molecular mechanism underlining the ripening process in banana remains to be further elucidated.

Overall, biological processes responsible for fruit ripening are complex. These data indicated that expression of the ACC oxidase gene modulates the fruit-ripening process through ethylene biosynthesis and signaling pathway. This is reflected by the down-regulation of a large number of genes related to the expression of *Mh-ACO1* or *Mh-ACO2* in concert with the down-regulation of genes related to ethylene signaling. In accord with results through high-throughput transcriptomic analysis, real time RT-PCR revealed comparable results for *Mh-ACO1* and *Mh-ACO2* at stages 1, 3, 5, and 7. Levels of expression were similar at stages 1, 3, 5, and 7 for both peel and pulp in the fruits. Interestingly, there seems to be a cascade gene expression pattern between the expression levels of *Mh-ACO1* and *Mh-ACO2*. For instance, in *Mh-ACO1* RNAi fruits, the gene-silencing effect leads to down-regulation of *Mh-ACO2* both in peel and pulp at stages 1, 3, 5, and 7 (Figure 8). However, in *Mh-ACO2* RNAi fruits, the gene-silencing effect resulted in an increased expression both in peel and pulp at stage 1 of ripening fruits. Strikingly, the expression levels of *Mh-ACO1* between peel and pulp are quite different for both *Mh-ACO1* and *Mh-ACO2* RNAi fruits. This correlates somehow with the suggestion made by Inaba et al. [26], where they proposed that ethylene biosynthesis in ripening banana fruit may be controlled negatively in the pulp tissue and positively in the peel tissue. To deepen the understanding of the biological mechanisms that mediate fruit ripening, the levels of expression and differences in expression kinetics spanning different stages of fruit ripening need to be investigated. Results of the present study demonstrate the potential for the use of RNA interference to better understand the mechanisms underlying fruit ripening.

## 4. Materials and Methods

### 4.1. Plant Materials

The plant material used in this research was *Musa* spp. cv. Pei-Chiao (AAA group, Cavendish subgroup). Untransformed (WT) and *Mh-ACO1* and *Mh-ACO2* RNAi transgenic banana plants were grown in a horticultural medium (soil:peat:sand, 1:1:1 by volume) contained in a 300 L plastic tank in an isolated greenhouse until flowering. The fruits of banana were harvested when the fruits had developed to highest flesh firmness at week 15 after inflorescence emergence. The detailed treatment of harvested fruits was performed as described in Hu et al. [42]. Each banana hand was separated into individual fingers and ripened at 20 °C naturally. Banana fruit ripening is divided into stages 1 to 8 by peel color according to the CSIRO Banana Ripening Guide [46]: all green; green with trace yellow; more green than yellow; more yellow than green; green tip; all yellow; yellow flecked with brown; and yellow with much brown, respectively. At the appropriate ripening stage, flesh tissue was frozen in liquid nitrogen and stored at −80 °C for extraction of total RNA.

### 4.2. Plasmid Construction and Banana Transformation

siRNA-expressing vector-based RNA interference has been applied for gene silencing targeting *Mh-ACO1* and *Mh-ACO2*. Two siRNA-expressing plasmid vectors were constructed using pGreen [47] as a backbone, in which siRNA-encoding regions were inserted downstream of the 2× *CaMV 35S* promoter. The regions used as antisense and sense fragments for the siRNA synthesis are indicated in Figure 2A. The first intron of *Mh-ACO1* [28] was used as a spacer to create stem loop-type siRNA-expression plasmid vectors driven by an enhanced double *CaMV 35S* promoter. The final siRNA-expressing vectors, pBI121-1AnS and pBI121-2AnS, were sequenced and used for banana transformation.

Banana transformation has been carried out with the banana embryogenic cells co-cultivated with *A. tumefaciens* strain LBA4404 harboring the binary plasmid pBI121-1AnS or pBI121-2AnS, as described in Hsu et al. [48] and modified by Chan et al. [49]. For somatic embryo development, the co-cultivated cells were transferred to the SH medium [50] supplemented with zeatin (0.05 mg·L^−1^), 2iP (0.2 mg·L^−1^), kinetin (0.1 mg·L^−1^), naphthalene acetic acid (0.2 mg·L^−1^), Cefotaxime (250 mg·L^−1^), and Geneticin (G418, Sigma; 50 mg·L^−1^). The germinating embryos were then transferred to MS medium [51] supplemented with benzyladenine (1 mg·L^−1^), gibberellic acid (0.1 mg·L^−1^), and Geneticin (100 mg·L^−1^) for plantlet development. The fully developed plants were transferred in the transgenic greenhouse and used for molecular analysis.

### 4.3. Southern Blot Analysis of Transgenic Banana Plants

Genomic DNA was extracted from non-transformed and putative transgenic plants as described by Dellaporta et al. [52]. Each genomic DNA sample (20 µg) was first digested with appropriate restriction enzymes, electrophoresed on a 0.7% (*w*/*v*) agarose gel, and transferred to a Hybond-N membrane (Amersham Pharmacia Biotech, Buckinghamshire, UK). Membranes were soaked overnight in hybridization solution (6× SSPE, 5× Denhardt’s Reagent, 0.5% SDS, 250 μg·mL^−1^ salmon sperm DNA, and 10% dextran sulfate) at 65 °C. DNA samples on the membranes were hybridized with probe labeled with ^32^P-dCTP using random-priming kit (Promega, Fitchburg, WI, USA). Then the membranes were washed twice for 15 min with Buffer I (2 × SSPE and 0.1% SDS) at room temperature, and twice for 15 min with Bbuffer II (1 × SSPE and 0.1% SDS) at 65 °C. Visual results were displayed by X-ray film (Kodak, USA) exposure.

### 4.4. Total RNA Extraction and Whole-Transcriptome Deep Sequencing

For transcriptome analysis, fruits harvested from Line 3 of *Mh-ACO1* silenced transgenic (As1), Line 6 of *Mh-ACO2* silenced transgenic (As2), and untransformed plants (WT) were collected at the same ripening stage (stage 3). In accordance with the manufacturer’s instructions, TRIzol Reagent (Invitrogen, USA) was used to extract total RNA from fruit samples. Degradation and contamination of RNA was monitored on 1% agarose gels. Purity of RNA was checked by the NanoPhotometer^®^ spectrophotometer (Implen, Inc., Westlake Village, CA, USA). RNA samples with an A_260_/A_280_ ratio from 1.9 to 2.1 were selected for analysis. Integrity of RNA was assessed by the Agilent 2100 Bioanalyzer (Agilent Technologies, Santa Clara, CA, USA) with the Algient RNA 6000 Nano Assay Kit. The input RNA sample weight was 3 μg. NEBNext^®^ Ultra™ RNA Library Prep Kit for Illumina^®^ (NEB, Ipswich, MA, USA) was used to generate sequencing libraries. AMPure XP system (Beckman Coulter, Brea, CA, USA) was used to purify the library fragments. Before conducting the PCR, 3 μL USER Enzyme (NEB, Ipswich, MA, USA) was used with size-selected, adaptor-ligated cDNA for 15 min at 37 °C followed by 5 min at 95 °C. AMPure XP system was used to purify PCR products. The Agilent 2100 Bioanalyzer system was used to assess library quality. The samples for transcriptome analysis were the mixture of equal amounts of RNA using HiSeq^TM^2500 Illumina as the sequencing platform for sequencing. Reads containing adapter, reads containing poly-N, and low quality reads from raw data were removed to obtain clean data (clean reads). The generated unigenes were analyzed by Blastx alignment search: (*E*-value < 10^‒5^) against protein databases nr, nt, and Swiss-Prot; *E*-value < 10^‒3^ for Blast alignment search against KOG protein databases; *E*-value < 10^‒10^ and *E*-value < 10^‒6^ for KEGG and GO databases, respectively.

Unigene sequences were functionally annotated and classified as the proteins with highest sequence similarity retrieved by Blastx from protein databases nr, Swiss-Prot, KEGG, and KOG. The best hits obtained from the Blastx against the nr database using Blastx2GO [34] were used to extract GO terms [38] based on the automated annotation of each unigene. These results were then sorted by GO categories using novogene script. Blastx was also used to align unique sequences to the Swiss-Prot database [36], PFAM [37], KOG [39], and KEGG [35] to predict possible functional classifications and molecular pathways.

Transcriptome reconstructed by Trinity is used as a reference [53]. Gene expression levels for each sample were estimated by RSEM [54]. Clean data were mapped onto the assembled transcriptome. Read count for each gene was then obtained after mapping. The level of gene expression was determined by calculating the number of fragments for each gene and then normalizing this to FPKM. [log2(Fold Change)] > 1 and q-value < 0.005 were used as the threshold to identify and compare differentially expressed genes (DEGs) in As1-vs-WT, As2-vs-WT and As1-vs-As2.

The KEGG enrichment scatter plot shows that the DEG enrichment analysis results in a KEGG pathway. The degree of KEGG enrichment is measured by Rich factor, q-value, and the number of genes enriched in this pathway. Rich factor refers to the ratio of the DEGs number in the pathway and the number of all genes annotated in the pathway. Q-value is the *p*-value after normalization, and its range is 0–1. The smaller the q-value is, the more significant the enrichment is.

### 4.5. Quantitative Real-Time PCR

The expression of selected genes involved in ethylene biosynthesis and signaling was quantified by real-time quantitative RT-PCRs (qRT-PCRs) using the RNA samples used for transcriptome analysis. Gene-specific primers used are listed in Table S6. Banana *Actin* gene was used as a normalizer. The PCR reaction was as follows: 2.5 μL 10× PCR buffer, 2 μL 2.5 mmol·L^−1^ dNTPs, 1 μL 5 μmol·L^−1^ forward and reverse primer, 0.2 μL Ex Taq (Takara, Japan), 0.1 μL cDNA template (contained 1 ng cDNA), and ddH_2_O added to 25 μL. The PCR conditions were: 95 °C for 3 min; 40 cycles for 95 °C for 15 s, 60 °C for 30 s and 72 °C for 20 s; 72 °C for 5 min; and a final 10 min extension step of 72 °C. The relative expression level was calculated by the 2^−∆∆*C*t^ method [55]. The relative mRNA abundances were visualized by heat maps. Relative transcript abundance was normalized with the wild-type banana and transformed into log2. Gene expression levels are indicated with a rainbow color scale from purple (very weakly expressed) to red (very strongly expressed). Mean data of two biological replicates were determined by three technical replicates.

### 4.6. R Programming for Heatmap Illustration

The expression patterns of genes involved in ethylene biosynthesis and signal transduction pathway in *Mh-ACO1* RNAi (As1) and *Mh-ACO2* RNAi (As2) transgenic banana plants were plotted on heat maps with the Gplots package [56] using R language (R Development Core Team, 2006). Of the 88,031 assembled contig IDs in WT, As1, and As2 unigene libraries, only those IDs that uniquely match to genes annotated in the Banana Genome Hub with pairwise identity of greater than 95% using BlastN are used to build the heat maps. The selected homologous genes’ IDs are as follows: SAMS—*S*-adenosylmethionine (SAM) synthetase; ACS—1-aminocyclopropane-1-carboxylic acid (ACC) synthase; ACO—ACC oxidase; ETR1—ethylene receptor; CTR1—Constitutive Triple Response 1; EIN2—Ethylene Insensitive 2; EIN3—Ethylene Insensitive 3; EBF—EIN3-binding F-box protein; ERF—ethylene responsive factor; RTE1—Reversion-to-Ethylene Sensitivity 1.

## Supplementary Materials

Supplementary materials can be found at www.mdpi.com/1422-0067/17/10/1632/s1.
