# Supplementary material for: Global Transcriptomic Analysis of Targeted Silencing of Two Paralogous ACC Oxidase Genes in Banana
Source: Int J Mol Sci. 2016 Sep 26;17(10):1632. doi: 10.3390/ijms17101632 (PMC5085665; doi:10.3390/ijms17101632)
Supplement: Supplementary file 1 [file ijms-17-01632-s001.pdf]

Supplementary Materials: Global Transcriptomic Analysis of Targeted Silencing of Two Paralogous ACC Oxidase Genes in Banana

Yan Xia, Chi Kuan, Chien-Hsiang Chiu, Xiao-Jing Chen, Yi-Yin Do and Pung-Ling Huang

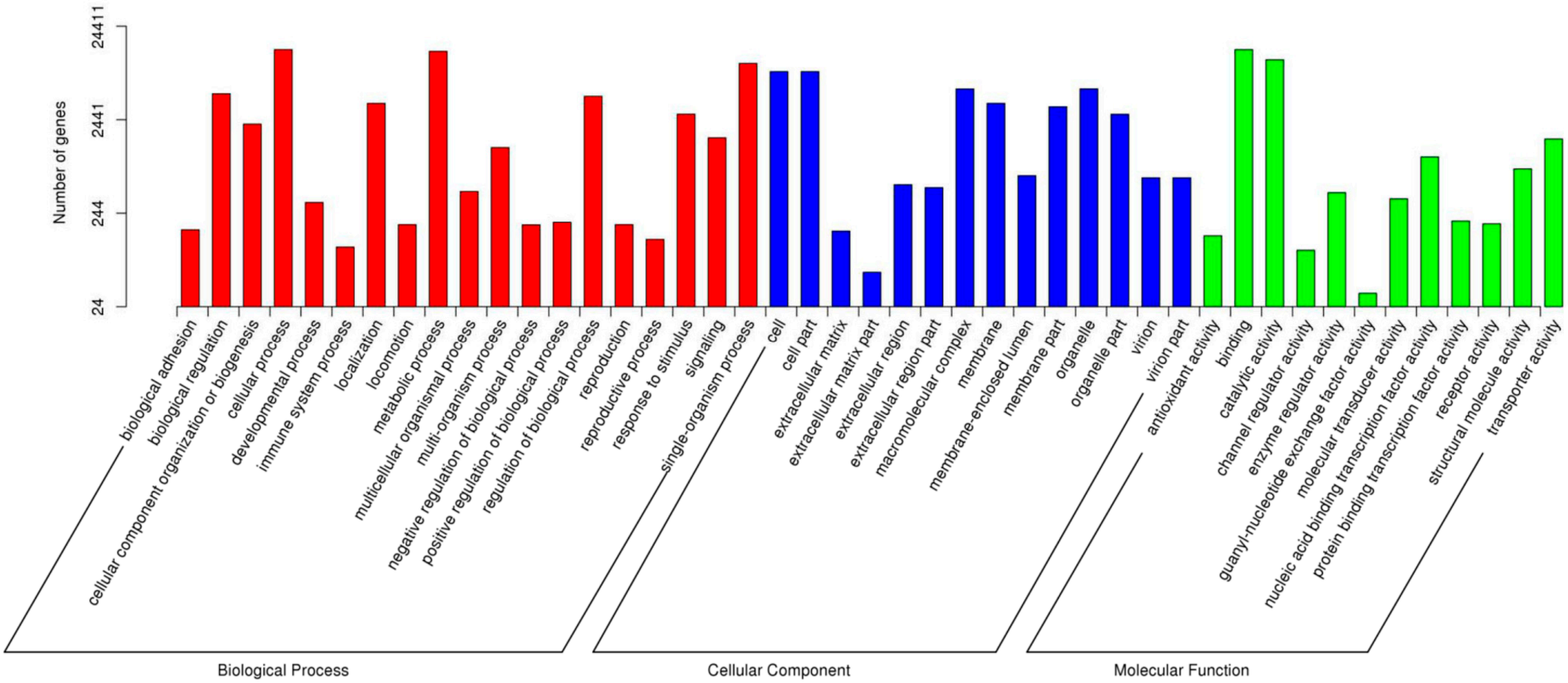

Figure S1. Gene Ontology (GO) terms of the transcriptomic sequences of banana samples of untransformed (WT), *Mh-ACO1 RNAi* (As1) and *Mh-ACO2 RNAi* (As2) transgenic plants.

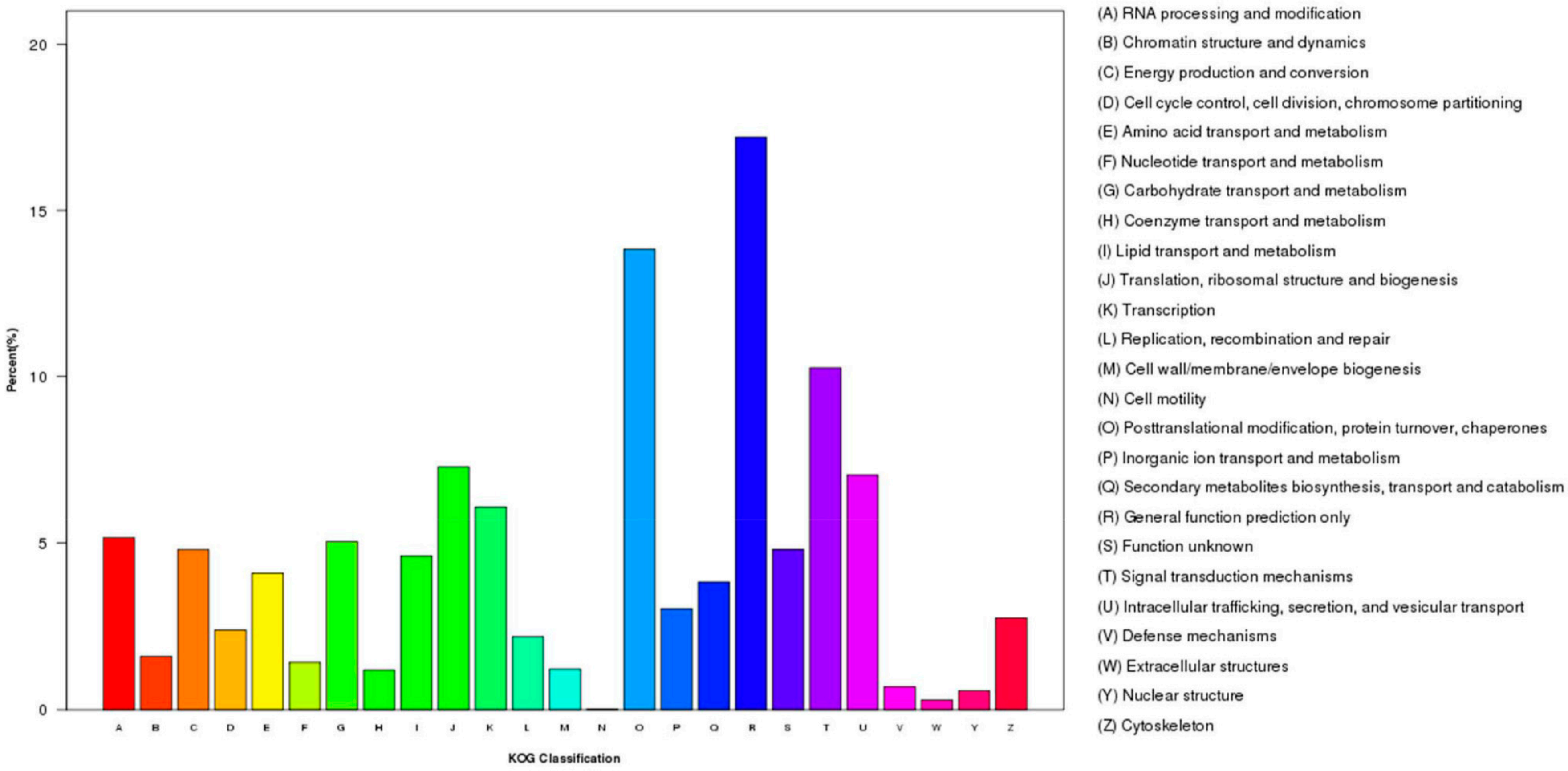

Figure S2. euKaryotic Orthologous Groups (KOG) classifications of transcriptomic sequences of banana samples of WT, As1, and As2.

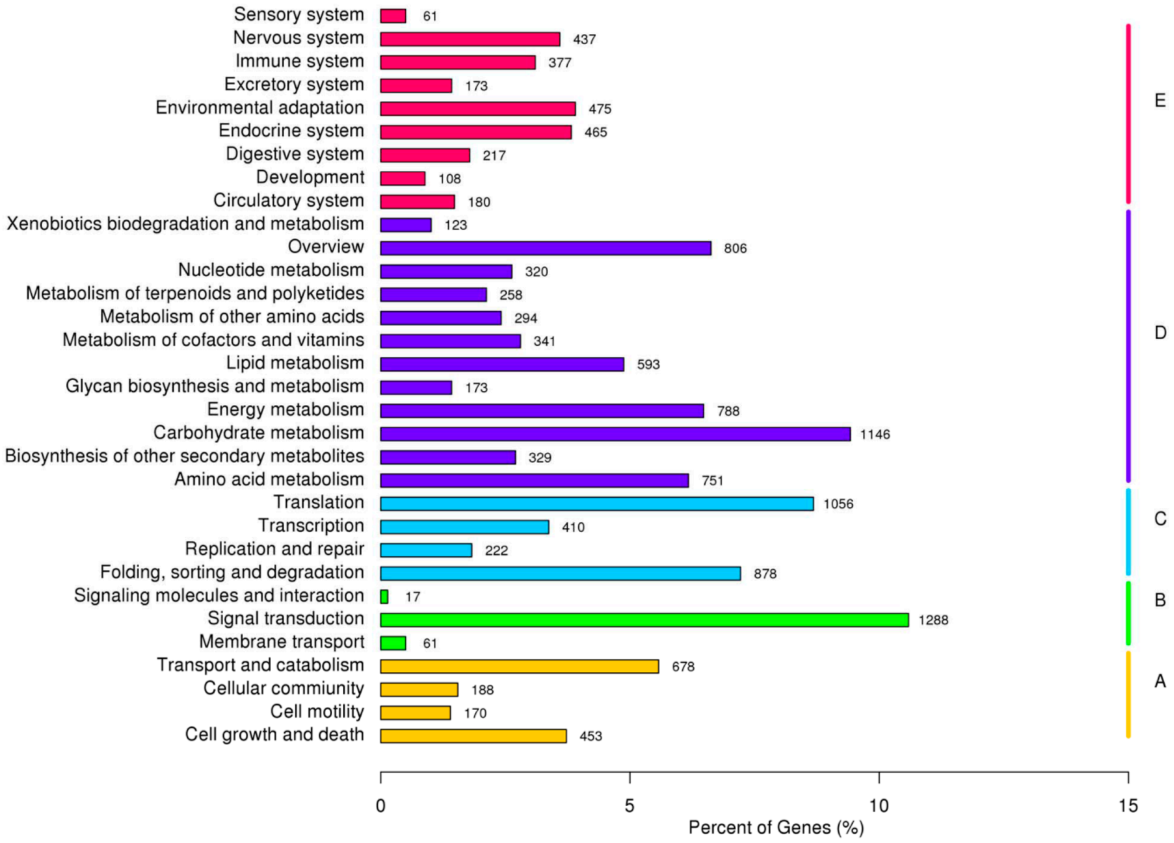

Figure S3. KEGG Classification.

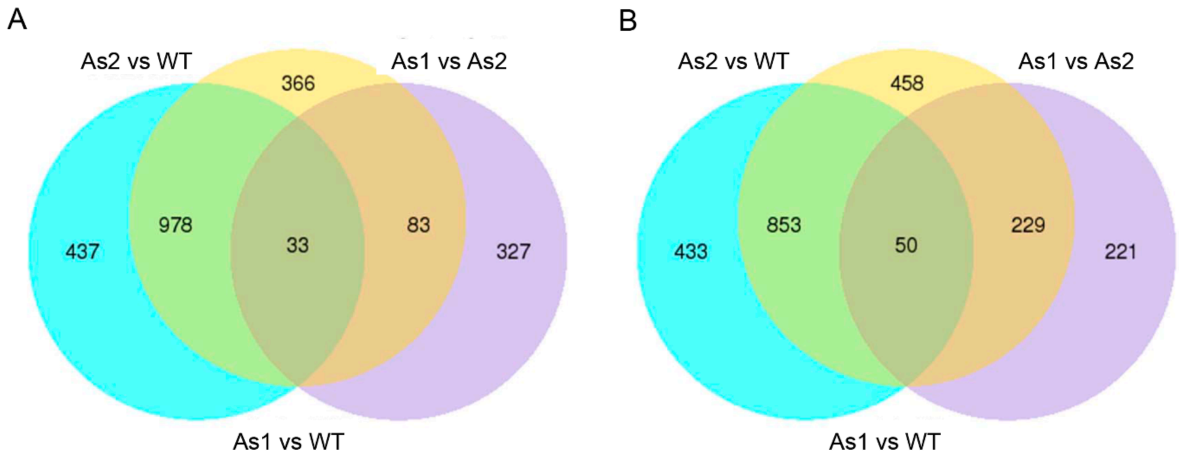

Figure S4. The Venn diagram of differentially expressed genes of banana samples of WT, As1, and As2.

Table S1. Gene ID, chromosomal distribution, and gene positions of the 18 ACC oxidase homologs on the *Musa acuminata* genome. Important protein domains in ACC oxidase, isopenicillin N synthase-like (IPR027443), non-haem dioxygenase N-terminal domain (IPR026992), and oxoglutarate/iron-dependent dioxygenase (IPR005123), are indicated as "V".

Gene ID	Chromosome Number	Gene Position		Length (nt)	Protein Domain		
		From	To		IPR024773	IPR026992	IPR005123
Ma01_p11540.1	chr01	8,344,279	8,345,473	1194	V	V	V
Ma03_p02700.1	chr03	1,836,668	1,838,116	1449	V	V	
Ma05_p09360.1	chr05	6,782,197	6,783,232	1036	V	V	V
Ma06_p02600.1	chr06	2,004,114	2,005,140	1027	V	V	V
Ma06_p14370.1	chr06	9,846,337	9,847,442	1106	V	V	V
Ma06_p14390.1	chr06	9,849,237	9,849,890	654	V	V	
Ma06_p14400.1	chr06	9,853,698	9,854,351	654	V	V	
Ma06_p14410.1	chr06	9,859,684	9,860,799	1116	V	V	V
Ma06_p14420.1	chr06	9,872,003	9,873,119	1117	V	V	V
Ma06_p14430.1	chr06	9,873,968	9,875,058	1091	V	V	V
Ma07_p19730.1	chr07	27,689,835	27,691,037	1203	V	V	V
Ma07_p26410.1	chr07	33,224,299	33,225,641	1343	V	V	
Ma08_p10790.1	chr08	7,910,807	7,922,354	11,548			
Ma10_p01130.1	chr10	4,027,514	4,031,525	4012	V	V	V
Ma10_p02410.1	chr10	7,867,969	7,868,244	276	V		V
Ma10_p16100.1	chr10	27,948,852	27,949,942	1091	V	V	V
Ma00_p04490.1	chrUn_random	38,627,956	38,629,261	1306	V	V	V
Ma00_p04770.1	chrUn_random	40,933,442	40,933,844	402	V		V

Table S2. Ethylene synthesis in untransformed (WT) and two transgenic *RNAi* banana lines targeting one of the *Mh-ACO1* and *Mh-ACO2* genes.

Plant	Day						
	5	10	15	20	25	30	35
WT	0.01 ± 0.01	0.04 ± 0.03	0.21 ± 0.08	1.75 ± 0.09	1.10 ± 0.15	0.52 ± 0.27	0.47 ± 0.19
Mh-ACO1-RNAi	0.01 ± 0.01	0.02 ± 0.01	0.05 ± 0.03	0.14 ± 0.08	0.15 ± 0.03	0.21 ± 0.14	0.17 ± 0.06
Mh-ACO2-RNAi	0.01 ± 0.01	0.01 ± 0.01	0.01 ± 0.01	0.02 ± 0.01	0.01 ± 0.01	0.02 ± 0.01	0.01 ± 0.01

Ethylene production of fruits from Line 3 of *Mh-ACO1-RNAi* (of the same line as shown in Figure 3) and Line 6 of *Mh-ACO2-RNAi* (of the same line as shown in Figure 4) transgenic banana was investigated. Ethylene generation was measured in natural ripening process by placing the banana fingers at mature green stage (day 1) in a sealed container at 20 °C. Measurements were determined as milliliters per gram per hour (± SE) with three biological replicates, each of which contained three technical replicates. Ethylene production was measured by injecting 1 mL of the head space gas in a gas chromatograph (CHROMPACK CP9001) fitted with flame ionization detector and an activated alumina (80–100 mesh) column.

Table S3. Genes used for Blast analysis in Figure 7. The gene annotations are obtained from Banana Genome Hub.

Gene Name Used in this Research	Gene ID in Banana Genome Hub	Gene Location	Start	End	Gene Annotation
SAMS	Ma01_p09270.1	chr01	6,702,694	6,703,878	Ma01_g09270~ *S*-adenosylmethionine synthase 2~ SAMS~ missing_completeness
SAMS	Ma01_p11680.1	chr01	8,473,991	8,474,486	Ma01_g11680~ *S*-adenosylmethionine synthase 2~ SAMS1~ missing_completeness
SAMS	Ma02_p09900.1	chr02	19,667,561	19,668,751	Ma02_g09900~ *S*-adenosylmethionine synthase 5~ SAMS~ complete
SAMS	Ma03_p08390.1	chr03	6,078,529	6,079,719	Ma03_g08390~ *S*-adenosylmethionine synthase~ SAMS~ missing_completeness
SAMS	Ma03_p12530.1	chr03	9,654,598	9,655,782	Ma03_g12530~ *S*-adenosylmethionine synthase~ SAMS~ missing_completeness
SAMS	Ma05_p23980.1	chr05	36,168,479	36,169,477	Ma05_g23980~ *S*-adenosylmethionine synthase~ SAMS2~ remnant
SAMS	Ma07_p02100.1	chr07	1,620,324	1,621,514	Ma07_g02100~ *S*-adenosylmethionine synthase~ SAMS~ missing_completeness
SAMS	Ma08_p06060.1	chr08	4,102,746	4,103,927	Ma08_g06060~ *S*-adenosylmethionine synthase~ SAMS~ missing_completeness
ACS	Ma01_p07800.1	chr01	5,646,180	5,647,826	Ma01_g07800~ 1-aminocyclopropane-1-carboxylate synthase CMA101~ ACS11~ complete
ACS	Ma02_p10500.1	chr02	20,041,654	20,043,371	Ma02_g10500~ 1-aminocyclopropane-1-carboxylate synthase 3~ ACC1~ complete
ACS	Ma03_p27050.1	chr03	30,526,857	30,528,463	Ma03_g27050~ 1-aminocyclopropane-1-carboxylate synthase 3~ ACS11~ complete
ACS	Ma04_p01260.1	chr04	1,111,602	1,113,254	Ma04_g01260~ 1-aminocyclopropane-1-carboxylate synthase 8~ ACS5~ complete
ACS	Ma04_p24230.1	chr04	26,284,739	26,286,400	Ma04_g24230~ 1-aminocyclopropane-1-carboxylate synthase CMA101~ ACS5~ complete
ACS	Ma04_p31490.1	chr04	31783,010	31,784,893	Ma04_g31490~ 1-aminocyclopropane-1-carboxylate synthase~ ACC1A~ complete
ACS	Ma04_p35640.1	chr04	34,320,367	34,322,186	Ma04_g35640~ 1-aminocyclopropane-1-carboxylate synthase~ ACC1A~ complete
ACS	Ma04_p37400.1	chr04	35,333,560	35,335,143	Ma04_g37400~ 1-aminocyclopropane-1-carboxylate synthase 7~ ACS5~ complete
ACS	Ma05_p08580.1	chr05	6,325,972	6,327,646	Ma05_g08580~ 1-aminocyclopropane-1-carboxylate synthase 3~ ACS5~ complete
ACS	Ma09_p19150.1	chr09	20,084,516	20,086,712	Ma09_g19150~ 1-aminocyclopropane-1-carboxylate synthase 2~ ACC1A~ complete
ACS	Ma10_p27510.1	chr10	34,765,916	34,767,472	Ma10_g27510~ 1-aminocyclopropane-1-carboxylate synthase 7~ ACS4~ complete
ACO	Ma01_p11540.1	chr01	8,344,279	8,345,473	Ma01_g11540~ 1-aminocyclopropane-1-carboxylate oxidase 1-like~ unknown_gene~ missing_functional_completeness
ACO	Ma03_p02700.1	chr03	1,836,668	1,838,116	Ma03_g02700~ 1-aminocyclopropane-1-carboxylate oxidase-like~ unknown_gene~ missing_functional_completeness
ACO	Ma05_p09360.1	chr05	6,782,197	6,783,232	Ma05_g09360~ 1-aminocyclopropane-1-carboxylate oxidase 1-like~ unknown_gene~ missing_functional_completeness
ACO	Ma06_p02600.1	chr06	2,004,114	2,005,140	Ma06_g02600~ 1-aminocyclopropane-1-carboxylate oxidase 1-like~ unknown_gene~ missing_functional_completeness
ACO	Ma06_p14370.1	chr06	9,846,337	9,847,442	Ma06_g14370~ 1-aminocyclopropane-1-carboxylate oxidase~ ACO2~ missing_completeness
ACO	Ma06_p14390.1	chr06	9,849,237	9,849,890	Ma06_g14390~ 1-aminocyclopropane-1-carboxylate oxidase-like~ unknown_gene~ missing_functional_completeness
ACO	Ma06_p14400.1	chr06	9,853,698	9,854,351	Ma06_g14400~ 1-aminocyclopropane-1-carboxylate oxidase-like~ unknown_gene~ missing_functional_completeness
ACO	Ma06_p14410.1	chr06	9,859,684	9,860,799	Ma06_g14410~ 1-aminocyclopropane-1-carboxylate oxidase-like~ unknown_gene~ missing_functional_completeness
ACO	Ma06_p14420.1	chr06	9,872,003	9,873,119	Ma06_g14420~ 1-aminocyclopropane-1-carboxylate oxidase-like~ unknown_gene~ missing_functional_completeness
ACO	Ma06_p14430.1	chr06	9,873,968	9,875,058	Ma06_g14430~ 1-aminocyclopropane-1-carboxylate oxidase-like~ unknown_gene~ missing_functional_completeness
ACO	Ma07_p19730.1	chr07	27,689,835	27,691,037	Ma07_g19730~ 1-aminocyclopropane-1-carboxylate oxidase-like~ unknown_gene~ missing_functional_completeness
ACO	Ma07_p26410.1	chr07	33,224,299	33,225,641	Ma07_g26410~ 1-aminocyclopropane-1-carboxylate oxidase~ unknown_gene~ missing_functional_completeness
ACO	Ma08_p10790.1	chr08	7,910,807	7,922,354	Ma08_g10790~ 1-aminocyclopropane-1-carboxylate oxidase-like~ unknown_gene~ missing_functional_completeness
ACO	Ma10_p01130.1	chr10	4,027,514	4,031,525	Ma10_g01130~ 1-aminocyclopropane-1-carboxylate oxidase homolog 3-like~ unknown_gene~ missing_functional_completeness
ACO	Ma10_p02410.1	chr10	7,867,969	7,868,244	Ma10_g02410~ Putative 1-aminocyclopropane-1-carboxylate oxidase homolog 4~ At1g06650~ fragment
ACO	Ma10_p16100.1	chr10	27,948,852	27,949,942	Ma10_g16100~ 1-aminocyclopropane-1-carboxylate oxidase~ ACO1~ complete

Table S3. *Cont.*

Gene Name Used in this Research	Gene ID in Banana Genome Hub	Gene Location	Start	End	Gene Annotation
ETR1	Ma03_p14190.1	chr03	11,332,289	11,335,060	Ma03_g14190~ ethylene receptor 2-like~ unknown_gene~ missing_functional_completeness
ETR1	Ma05_p00120.1	chr05	86,757	92,356	Ma05_g00120~ ethylene receptor-like, transcript variant X2~ unknown_gene~ missing_functional_completeness
ETR1	Ma05_p00120.2	chr05	86,757	92,356	Ma05_g00120~ ethylene receptor-like, transcript variant X2~ unknown_gene~ missing_functional_completeness
ETR1	Ma05_p23710.1	chr05	35,863,097	35,872,242	Ma05_g23710~ Ethylene receptor~ ETR2~ missing_completeness
ETR1	Ma06_p35320.1	chr06	35,219,142	35,222,060	Ma06_g35320~ ethylene receptor 2-like~ unknown_gene~ missing_functional_completeness
ETR1	Ma11_p02040.1	chr11	1,456,185	1,459,604	Ma11_g02040~ Putative Ethylene receptor 2~ ETR2~ missing_completeness
ETR1	Ma11_p16580.1	chr11	22,077,127	22,079,319	Ma11_g16580~ Ethylene receptor~ ETR1~ fragment
ETR1	Ma11_p16590.1	chr11	22,079,370	22,079,522	Ma11_g16590~ Ethylene receptor 1~ ETR1~ fragment
ETR1	Ma11_p18980.1	chr11	23,963,608	23,969,213	Ma11_g18980~ Ethylene receptor~ ETR2~ complete
CTR1	Ma08_p07460.1	chr08	5,106,637	5,117,078	Ma08_g07460~ Serine/threonine-protein kinase CTR1~ shkC~ complete
CTR1	Ma11_p13050.1	chr11	17,199,697	17,211,108	Ma11_g13050~ serine/threonine-protein kinase CTR1-like~ unknown_gene~ missing_functional_completeness
CTR1	Ma11_p13160.1	chr11	17,421,645	17,433,502	Ma11_g13160~ Serine/threonine-protein kinase CTR1~ DDB_G0267514~ missing_completeness
CTR1	Ma11_p23960.1	chr11	27,125,545	27,135,808	Ma11_g23960~ serine/threonine-protein kinase CTR1~ unknown_gene~ missing_functional_completeness
EIN2	Ma06_p32860.1	chr06	33,687,876	33,695,858	Ma06_g32860~ ethylene-insensitive protein 2-like~ unknown_gene~ missing_functional_completeness
EIN2	Ma07_p01290.1	chr07	993,258	1,003,071	Ma07_g01290~ ethylene-insensitive protein 2-like, transcript variant X1~ unknown_gene~ missing_functional_completeness
EIN2	Ma07_p01290.2	chr07	994,036	1,003,071	Ma07_g01290~ ethylene-insensitive protein 2-like, transcript variant X1~ unknown_gene~ missing_functional_completeness
EIN2	Ma09_p09950.1	chr09	6,800,065	6,807,685	Ma09_g09950~ ethylene-insensitive protein 2-like, transcript variant X2~ unknown_gene~ missing_functional_completeness
EIN2	Ma09_p09950.2	chr09	6,800,065	6,807,685	Ma09_g09950~ ethylene-insensitive protein 2-like, transcript variant X2~ unknown_gene~ missing_functional_completeness
EIN2	Ma09_p09950.3	chr09	6,800,065	6,807,685	Ma09_g09950~ ethylene-insensitive protein 2-like, transcript variant X2~ unknown_gene~ missing_functional_completeness
EIN2	Ma09_p09950.4	chr09	6,800,065	6,807,685	Ma09_g09950~ ethylene-insensitive protein 2-like, transcript variant X2~ unknown_gene~ missing_functional_completeness
EIN2	Ma09_p09950.5	chr09	6,800,065	6,807,685	Ma09_g09950~ ethylene-insensitive protein 2-like, transcript variant X2~ unknown_gene~ missing_functional_completeness
EIN3	Ma02_p13450.1	chr02	21,833,942	21,835,756	Ma02_g13450~ Protein ETHYLENE INSENSITIVE 3~ EIL3~ missing_completeness
EIN3	Ma03_p09300.1	chr03	6,856,313	6,858,043	Ma03_g09300~ ETHYLENE INSENSITIVE 3-like 1 protein~ unknown_gene~ missing_functional_completeness
EIN3	Ma03_p10900.1	chr03	8,234,550	8,236,403	Ma03_g10900~ ETHYLENE INSENSITIVE 3-like 3 protein, transcript variant X2~ unknown_gene~ missing_functional_completeness
EIN3	Ma03_p10900.2	chr03	8234550	8,236,403	Ma03_g10900~ ETHYLENE INSENSITIVE 3-like 3 protein, transcript variant X2~ unknown_gene~ missing_functional_completeness
EIN3	Ma05_p30040.1	chr05	40,578,590	40,580,473	Ma05_g30040~ Putative Protein ETHYLENE INSENSITIVE 3~ EIL3~ missing_completeness
EIN3	Ma06_p00860.1	chr06	717,474	719,060	Ma06_g00860~ protein ETHYLENE INSENSITIVE 3-like~ unknown_gene~ missing_functional_completeness
EIN3	Ma06_p17470.1	chr06	11,866,439	11,868,346	Ma06_g17470~ ETHYLENE INSENSITIVE 3-like 1 protein~ unknown_gene~ missing_functional_completeness
EIN3	Ma06_p33860.1	chr06	34,325,893	34,327,740	Ma06_g33860~ Protein ETHYLENE INSENSITIVE 3~ EIL3~ missing_completeness
EIN3	Ma07_p17960.1	chr07	22,743,283	22,744,938	Ma07_g17960~ protein ETHYLENE INSENSITIVE 3-like~ unknown_gene~ missing_functional_completeness
EIN3	Ma08_p22320.1	chr08	35,940,216	35,942,120	Ma08_g22320~ Putative Protein ETHYLENE INSENSITIVE 3~ EIL3~ missing_completeness
EIN3	Ma08_p26350.1	chr08	38,927,038	38,928,888	Ma08_g26350~ ETHYLENE INSENSITIVE 3-like 3 protein~ unknown_gene~ missing_functional_completeness
EIN3	Ma09_p16910.1	chr09	12,406,725	12,408,593	Ma09_g16910~ ETHYLENE INSENSITIVE 3-like 1 protein, transcript variant X2~ unknown_gene~ missing_functional_completeness
EIN3	Ma09_p16910.2	chr09	12,406,725	12,408,593	Ma09_g16910~ ETHYLENE INSENSITIVE 3-like 1 protein, transcript variant X2~ unknown_gene~ missing_functional_completeness
EIN3	Ma09_p28310.1	chr09	39,112,476	39,114,308	Ma09_g28310~ ETHYLENE INSENSITIVE 3-like 3 protein~ unknown_gene~ missing_functional_completeness

Table S3. *Cont.*

Gene Name Used in this Research	Gene ID in Banana Genome Hub	Gene Location	Start	End	Gene Annotation
EIN3	Ma10_p12970.1	chr10	25,924,761	25,926,674	Ma10_g12970~ ETHYLENE INSENSITIVE 3-like 1 protein~ unknown_gene~ missing_functional_completeness
EBF	Ma01_p11960.1	chr01	8,660,059	8,661,906	Ma01_g11960~ EIN3-binding F-box protein 1-like~ unknown_gene~ missing_functional_completeness
EBF	Ma02_p17690.1	chr02	24,504,401	24,506,877	Ma02_g17690~ EIN3-binding F-box protein 1-like~ unknown_gene~ missing_functional_completeness
EBF	Ma03_p12220.1	chr03	9,453,794	9,455,976	Ma03_g12220~ EIN3-binding F-box protein 1-like~ unknown_gene~ missing_functional_completeness
EBF	Ma04_p37200.1	chr04	35,230,659	35,233,155	Ma04_g37200~ Putative EIN3-binding F-box protein 1~ EBF1~ missing_completeness
EBF	Ma06_p06840.1	chr06	4,904,057	4,906,677	Ma06_g06840~ Putative EIN3-binding F-box protein 1~ EBF1~ missing_completeness
EBF	Ma07_p18840.1	chr07	26,777,953	26,779,767	Ma07_g18840~ EIN3-binding F-box protein 1-like~ unknown_gene~ missing_functional_completeness
EBF	Ma09_p29140.1	chr09	39,678,005	39,680,621	Ma09_g29140~ Putative EIN3-binding F-box protein 1~ EBF1~ missing_completeness
EBF	Ma10_p26870.1	chr10	34,360,983	34,362,989	Ma10_g26870~ Putative EIN3-binding F-box protein 1~ EBF1~ missing_completeness
RTE1	Ma02_p19360.1	chr02	25,541,962	25,542,704	Ma02_g19360~ protein RTE1-HOMOLOG, transcript variant X1~ unknown_gene~ missing_functional_completeness
RTE1	Ma02_p19360.2	chr02	25,541,962	25,542,704	Ma02_g19360~ protein RTE1-HOMOLOG, transcript variant X1~ unknown_gene~ missing_functional_completeness
ERF	Ma02_p09170.1	chr02	19,300,352	19,301,026	Ma02_g09170~ ethylene-responsive transcription factor ERF018-like~ unknown_gene~ missing_functional_completeness
ERF	Ma02_p09180.1	chr02	19,302,866	19,303,396	Ma02_g09180~ ethylene-responsive transcription factor ERF017-like~ unknown_gene~ missing_functional_completeness
ERF	Ma02_p12320.1	chr02	21,093,554	21,094,336	Ma02_g12320~ Putative Ethylene-responsive transcription factor ERF105~ ERF2~ complete
ERF	Ma02_p13710.1	chr02	21,975,296	21,975,877	Ma02_g13710~ ethylene-responsive transcription factor ERF026-like~ unknown_gene~ missing_functional_completeness
ERF	Ma02_p13870.1	chr02	22,062,045	22,062,686	Ma02_g13870~ Putative Ethylene-responsive transcription factor ERF034~ unknown_gene~ complete
ERF	Ma02_p22690.1	chr02	27,954,192	27,954,758	Ma02_g22690~ Putative Ethylene-responsive transcription factor ERF021~ unknown_gene~ complete
ERF	Ma03_p00160.1	chr03	215,283	215,648	Ma03_g00160~ Ethylene-responsive transcription factor ERF098~ unknown_gene~ complete
ERF	Ma03_p02200.1	chr03	1,500,398	1,501,324	Ma03_g02200~ ethylene-responsive transcription factor ERF113-like~ unknown_gene~ missing_functional_completeness
ERF	Ma03_p04220.1	chr03	2,765,197	2,766,309	Ma03_g04220~ Putative Ethylene-responsive transcription factor ERF058~ unknown_gene~ complete
ERF	Ma03_p05830.1	chr03	4,009,891	4,010,418	Ma03_g05830~ ethylene-responsive transcription factor ERF016-like~ unknown_gene~ missing_functional_completeness
ERF	Ma03_p08080.1	chr03	5,826,013	5,826,652	Ma03_g08080~ Putative Ethylene-responsive transcription factor ERF071~ unknown_gene~ complete
ERF	Ma03_p23580.1	chr03	28,121,985	28,123,247	Ma03_g23580~ ethylene-responsive transcription factor ERF112~ unknown_gene~ complete
ERF	Ma04_p02230.1	chr04	1,938,458	1,938,995	Ma04_g02230~ ethylene-responsive transcription factor ERF003-like~ unknown_gene~ missing_functional_completeness
ERF	Ma04_p05550.1	chr04	4,137,320	4,137,772	Ma04_g05550~ Ethylene-responsive transcription factor ERF098~ ERF2~ complete
ERF	Ma04_p12050.1	chr04	8,621,875	8,622,258	Ma04_g12050~ Ethylene-responsive transcription factor ERF098~ unknown_gene~ complete
ERF	Ma04_p21170.1	chr04	23,749,491	23,750,273	Ma04_g21170~ Putative Ethylene-responsive transcription factor ERF105~ ERF1~ complete
ERF	Ma04_p23660.1	chr04	25,762,553	25,763,221	Ma04_g23660~ Ethylene-responsive transcription factor ERF025~ unknown_gene~ complete
ERF	Ma04_p24880.1	chr04	26,767,002	26,767,463	Ma04_g24880~ Putative Ethylene-responsive transcription factor ERF061~ unknown_gene~ fragment
ERF	Ma04_p31090.1	chr04	31,505,634	31,506,347	Ma04_g31090~ Putative Ethylene-responsive transcription factor ERF034~ unknown_gene~ complete
ERF	Ma04_p31420.1	chr04	31,731,853	31,732,551	Ma04_g31420~ ethylene-responsive transcription factor ERF027~ unknown_gene~ missing_functional_completeness
ERF	Ma04_p32650.1	chr04	32,591,395	32,598,458	Ma04_g32650~ Putative Ethylene-responsive transcription factor ERF054~ unknown_gene~ remnant
ERF	Ma04_p32660.1	chr04	32,613,955	32,615,238	Ma04_g32660~ Putative Ethylene-responsive transcription factor ERF053~ unknown_gene~ complete
ERF	Ma04_p33540.1	chr04	33,111,422	33,112,174	Ma04_g33540~ Ethylene-responsive transcription factor ERF034~ unknown_gene~ complete
ERF	Ma04_p35580.1	chr04	34,288,896	34,289,588	Ma04_g35580~ Ethylene-responsive transcription factor ERF025~ unknown_gene~ complete
ERF	Ma05_p04180.1	chr05	3,132,607	3,133,332	Ma05_g04180~ ethylene-responsive transcription factor ERF084~ unknown_gene~ missing_functional_completeness
ERF	Ma05_p04880.1	chr05	3,754,066	3,754,641	Ma05_g04880~ Putative Ethylene-responsive transcription factor ERF017~ unknown_gene~ complete
ERF	Ma05_p09130.1	chr05	6,665,451	6,666,203	Ma05_g09130~ Putative Ethylene-responsive transcription factor ERF061~ unknown_gene~ complete
ERF	Ma05_p11800.1	chr05	8,628,946	8,630,058	Ma05_g11800~ Putative Ethylene-responsive transcription factor ERF053~ unknown_gene~ complete

Table S3. *Cont.*

Gene Name Used in this Research	Gene ID in Banana Genome Hub	Gene Location	Start	End	Gene Annotation
ERF	Ma05_p12300.1	chr05	8,914,898	8,915,563	Ma05_g12300~ Putative Ethylene-responsive transcription factor ERF105~ unknown_gene~ complete
ERF	Ma05_p13470.1	chr05	9,779,322	9,780,062	Ma05_g13470~ Putative Ethylene-responsive transcription factor ERF034~ unknown_gene~ complete
ERF	Ma05_p15250.1	chr05	11,339,733	11,340,221	Ma05_g15250~ Putative Ethylene-responsive transcription factor ERF034~ unknown_gene~ complete
ERF	Ma05_p23760.1	chr05	35,907,824	35,908,573	Ma05_g23760~ Putative Ethylene-responsive transcription factor ERF071~ ERF056~ complete
ERF	Ma05_p24610.1	chr05	36,854,578	36,855,144	Ma05_g24610~ Ethylene-responsive transcription factor ERF011~ unknown_gene~ complete
ERF	Ma05_p25230.1	chr05	37,221,124	37,221,691	Ma05_g25230~ ethylene-responsive transcription factor ERF003-like~ unknown_gene~ missing_functional_completeness
ERF	Ma06_p03990.1	chr06	2,873,588	2,874,253	Ma06_g03990~ Ethylene-responsive transcription factor ERF071~ ERF4~ complete
ERF	Ma06_p09740.1	chr06	6,776,752	6,778,960	Ma06_g09740~ ethylene-responsive transcription factor ERF114-like~ unknown_gene~ missing_functional_completeness
ERF	Ma06_p13190.1	chr06	9,041,498	9,042,193	Ma06_g13190~ ethylene-responsive transcription factor ERF018-like~ unknown_gene~ missing_functional_completeness
ERF	Ma07_p05490.1	chr07	3,983,363	3,983,836	Ma07_g05490~ Ethylene-responsive transcription factor ERF010~ unknown_gene~ complete
ERF	Ma07_p06960.1	chr07	5,038,216	5,038,926	Ma07_g06960~ Putative Ethylene-responsive transcription factor ERF105~ ERF2~ complete
ERF	Ma07_p07020.1	chr07	5,098,939	5,099,661	Ma07_g07020~ Ethylene-responsive transcription factor ERF038~ unknown_gene~ complete
ERF	Ma07_p11230.1	chr07	8,361,763	8,362,437	Ma07_g11230~ ethylene-responsive transcription factor ERF017-like~ unknown_gene~ missing_functional_completeness
ERF	Ma07_p16410.1	chr07	13,292,460	13,293,088	Ma07_g16410~ ethylene-responsive transcription factor ERF003-like~ unknown_gene~ missing_functional_completeness
ERF	Ma07_p20590.1	chr07	28,467,594	28,468,721	Ma07_g20590~ Putative Ethylene-responsive transcription factor ERF058~ ERF053~ complete
ERF	Ma07_p20810.1	chr07	28,815,750	28,816,392	Ma07_g20810~ ethylene-responsive transcription factor ERF003-like~ unknown_gene~ missing_functional_completeness
ERF	Ma08_p10740.1	chr08	7,873,707	7,874,228	Ma08_g10740~ Putative Ethylene-responsive transcription factor ERF105~ unknown_gene~ complete
ERF	Ma08_p13160.1	chr08	10,450,008	10,451,540	Ma08_g13160~ Putative Ethylene-responsive transcription factor ERF054~ ERF057~ complete
ERF	Ma08_p16090.1	chr08	17,004,034	17,004,926	Ma08_g16090~ ethylene-responsive transcription factor ERF112~ infB~ complete
ERF	Ma08_p19170.1	chr08	32,971,638	32,972,135	Ma08_g19170~ Putative Ethylene-responsive transcription factor ERF021~ unknown_gene~ complete
ERF	Ma08_p30660.1	chr08	41,845,752	41,846,837	Ma08_g30660~ ethylene-responsive transcription factor ERF086~ unknown_gene~ missing_functional_completeness
ERF	Ma09_p13280.1	chr09	8,963,967	8,964,510	Ma09_g13280~ ethylene-responsive transcription factor ERF003-like~ unknown_gene~ missing_functional_completeness
ERF	Ma10_p03020.1	chr10	10,641,323	10,641,892	Ma10_g03020~ ethylene-responsive transcription factor ERF017-like~ unknown_gene~ missing_functional_completeness
ERF	Ma10_p12340.1	chr10	25,497,038	25,498,159	Ma10_g12340~ ethylene-responsive transcription factor ERF113-like~ unknown_gene~ missing_functional_completeness
ERF	Ma10_p17490.1	chr10	28,860,811	28,861,371	Ma10_g17490~ ethylene-responsive transcription factor ERF017-like~ unknown_gene~ missing_functional_completeness
ERF	Ma10_p19470.1	chr10	30,008,585	30,009,232	Ma10_g19470~ Putative Ethylene-responsive transcription factor ERF012~ unknown_gene~ complete
ERF	Ma10_p24030.1	chr10	32,750,731	32,751,355	Ma10_g24030~ ethylene-responsive transcription factor ERF003-like~ unknown_gene~ missing_functional_completeness
ERF	Ma10_p25810.1	chr10	33,775,756	33,776,295	Ma10_g25810~ ethylene-responsive transcription factor ERF017-like~ unknown_gene~ missing_functional_completeness
ERF	Ma10_p30050.1	chr10	36,456,516	3,645,7274	Ma10_g30050~ Ethylene-responsive transcription factor ERF071~ unknown_gene~ complete
ERF	Ma11_p03090.1	chr11	2,278,764	2,279,435	Ma11_g03090~ Putative Ethylene-responsive transcription factor ERF024~ unknown_gene~ complete
ERF	Ma11_p09230.1	chr11	7,366,239	7,367,190	Ma11_g09230~ ethylene-responsive transcription factor ERF113-like~ unknown_gene~ missing_functional_completeness
ERF	Ma11_p09750.1	chr11	9,021,418	9,021,999	Ma11_g09750~ Putative Ethylene-responsive transcription factor ERF021~ unknown_gene~ complete
ERF	Ma11_p14400.1	chr11	19,689,433	19,690,141	Ma11_g14400~ Ethylene-responsive transcription factor ERF071~ unknown_gene~ complete
ERF	Ma11_p14800.1	chr11	20,491,479	20,491,856	Ma11_g14800~ ethylene-responsive transcription factor ERF038-like~ unknown_gene~ missing_functional_completeness
ERF	Ma11_p14850.1	chr11	20,546,929	20,547,820	Ma11_g14850~ ethylene-responsive transcription factor ERF039-like~ unknown_gene~ missing_functional_completeness
ERF	Ma11_p18300.1	chr11	23,389,488	23,390,309	Ma11_g18300~ ethylene-responsive transcription factor ERF069-like~ unknown_gene~ missing_functional_completeness
ERF	Ma11_p19360.1	chr11	24,258,665	24,259,438	Ma11_g19360~ Putative Ethylene-responsive transcription factor ERF034~ unknown_gene~ complete

Table S4. Fragments per kilobase of transcript per million mapped reads (FPKM) value applied for heat map generation in Figure 7.

Gene Name Used in this Research	Contig ID	FPKM_WT	FPKM_As1	FPKM_As2
SAMS	c2701_g1	38.98	59.01	24.15
SAMS	c11230_g1	8.97	16.02	6.37
SAMS	c18290_g1	4.93	22.56	13
SAMS	c28686_g1	21.53	26.45	8.91
SAMS	c30914_g1	26.45	31.82	17.37
SAMS	c33799_g1	218	79.84	112.59
SAMS	c37395_g1	78.35	108.32	140.51
SAMS	c37395_g2	92.52	123.35	133.76
SAMS	c46690_g2	411.72	44.45	116.67
SAMS	c51749_g2	1787.16	1212.28	1675.39
SAMS	c68588_g1	2.98	0	0
SAMS	c75750_g1	0	0	0
ACS	c35665_g1	11.11	343.87	375.84
ACO	c7774_g1	0	4.35	0
ACO	c11205_g1	0	3.89	0
ACO	c11207_g1	0.07	3.26	10.47
ACO	c32789_g1	0.92	1.61	2.49
ACO	c34457_g1	152.35	175.89	159.17
ACO	c35493_g1	0.56	1.15	0.66
ACO	c35493_g2	1.65	1.16	1.27
ACO	c35892_g1	7362.54	5231.07	11379.37
ACO	c38034_g1	17.1	10.5	11.8
ACO	c51532_g1	27.02	76	66.39
ACO	c51532_g2	0	9.37	1.05
ACO	c51532_g3	0.78	6.32	3.08
ACO	c56565_g1	0	1.44	2.72
ACO	c74673_g1	0	1.53	0.71
ETR1	c6101_g1	2.79	2.66	2.17
ETR1	c22899_g1	2.76	1.13	0.53
ETR1	c29444_g1	2.96	1.32	0.63
ETR1	c40150_g1	23.93	24.68	17.59
ETR1	c53493_g1	54.53	85.58	63.93
ETR1	c53686_g2	25.11	40.5	42.28
ETR1	c84775_g1	0.25	0.62	0.6
CTR1	c37237_g1	19.63	14.08	13.74
CTR1	c44831_g1	20.86	9.09	4.68
CTR1	c51824_g1	7.74	9.12	7.26
CTR1	c54936_g1	107.92	17.69	12.04
EIN2	c53048_g1	15.69	14.87	7.12
EIN3	c20815_g1	22.47	47.42	53.2
EIN3	c24165_g1	44.02	27.12	13.9
EIN3	c24406_g1	19.85	35.14	36.09
EIN3	c26480_g1	4.66	4.23	3.16
EIN3	c30037_g1	37.51	16.13	16.67

Table S4. *Cont.*

Gene Name Used in this Research	Contig ID	FPKM_WT	FPKM_As1	FPKM_As2
EIN3	c30037_g2	38.22	4.91	17.01
EIN3	c37848_g1	72.26	31.09	29
EIN3	c43060_g1	4.9	3.29	3.66
EIN3	c44692_g1	45.98	26.26	21
EIN3	c44692_g2	67.15	26.64	14.06
EIN3	c51539_g1	80.75	42.1	23.82
EIN3	c51539_g2	16.2	38.02	25.68
EIN3	c52920_g1	10.73	8.5	10.28
EIN3	c52920_g2	5.52	4.47	5.37
EIN3	c52920_g3	7.24	7.37	10.23
EIN3	c53837_g1	335.35	326.69	214.95
EIN3	c53837_g2	52.71	25.83	18.62
EIN3	c56037_g1	0.46	0.56	1.05
EIN3	c69792_g1	6.61	0	0
EIN3	c74777_g1	1.28	1.32	0.25
EIN3	c83439_g1	3.92	2.13	5.01
EBF	c24910_g1	5.02	5.77	4.97
EBF	c30250_g1	34.85	8.63	14.72
EBF	c33234_g1	15.26	13.26	11.94
EBF	c35643_g1	28.94	16.68	10.08
EBF	c39553_g1	34.04	12.67	11.43
EBF	c46363_g1	15.04	20.19	16
EBF	c46363_g2	14.63	23.57	22.91
EBF	c53655_g1	27.82	12.86	19.74
EBF	c53655_g2	61.62	91.66	85.02
EBF	c53655_g3	0	4.91	0
EBF	c53655_g4	13.6	31.36	13.56
EBF	c53655_g5	32.06	25.73	12.4
EBF	c59478_g1	52.46	13.04	22.69
RTE1	c38845_g1	51.33	3.73	7.26
ERF	c9581_g1	1.81	1.04	1.29
ERF	c10566_g1	1.67	0.8	1.33
ERF	c11063_g1	0	2.72	0
ERF	c18615_g1	0.35	0.42	3.62
ERF	c24096_g1	1.09	1.4	1.16
ERF	c25845_g1	4.36	4.02	1.8
ERF	c31216_g1	4.59	46.67	13.56
ERF	c35908_g1	2.86	10.62	23.32
ERF	c37998_g1	2.32	62.46	28.68
ERF	c39615_g1	0.28	13.44	33.54
ERF	c40349_g1	2.34	2.52	2.44
ERF	c40808_g1	0.3	11.63	16.87
ERF	c41484_g1	103.39	65.43	51.87
ERF	c42280_g1	4.08	6.06	2.34
ERF	c42329_g1	4	1.14	2.14
ERF	c42347_g1	23.97	6.87	5.43

Table S4. *Cont.*

Gene Name Used in this Research	Contig ID	FPKM_WT	FPKM_As1	FPKM_As2
ERF	c44459_g1	1.85	5.8	2.67
ERF	c44947_g1	12.71	4.42	4.11
ERF	c44947_g2	23.2	6.19	2.84
ERF	c45398_g2	2.78	3.18	2.85
ERF	c50847_g1	10.76	53.32	20.78
ERF	c51156_g2	61.22	53.99	18.59
ERF	c56220_g1	0.58	2.13	0.34
ERF	c56460_g1	0	0	1.09
ERF	c57801_g1	1.89	0.77	0.73
ERF	c58447_g1	0	3.6	3.35
ERF	c58987_g1	0	0	8.25
ERF	c62187_g1	0	1.24	2.36
ERF	c65096_g1	0.84	0	5.62
ERF	c68870_g1	1.39	1.66	5.97
ERF	c70175_g1	0.83	1.01	0
ERF	c71060_g1	0	4.73	0.0001
ERF	c71278_g1	0	2.63	3.32
ERF	c71548_g1	0	0.78	1.25
ERF	c71809_g1	0.66	0.81	1.53
ERF	c87429_g1	0	2.04	0.94

Table S5. Relative mRNA abundances based on qRT-PCR data in *Mh-ACO1 RNAi* (As1) and *Mh-ACO2 RNAi* (As2) transgenic and untransformed (WT) banana fruits.

Gene Name	Ripening Stage	Peel			Pulp		
		WT	As1	As2	WT	As1	As2
Mh-ACS1	1	1.000342	5.605955	1.312929602	1.016021601	2.016886	0.30381
	3	33.93106	6.154716	143.1319173	210.0327282	0.752604	199.5915
	5	237.6897	2.110378	281.0408658	202.9815409	5.774466	391.1312
	7	445.8393	504.4911	151.9006009	495.8166616	1370.892	1150.437
Mh-ACO1	1	1.00234	1.144339	3.555770497	1.002454731	5.378275	2.942405
	3	0.10752	0.142465	0.096496782	6.609935143	0.604636	13.2013
	5	0.823721	0.014481	0.367904862	1.446242589	1.529717	0.14027
	7	0.097048	0.175516	0.124602083	9.496729744	0.941501	2.805201
Mh-ACO2	1	1.000208	0.032628	2.628976465	1.009075614	0.186668	0.197084
	3	33.90554	3.988296	33.78692529	2.125343965	0.61629	1.818054
	5	52.76232	4.010951	44.37925509	2.252178477	0.403475	0.629347
	7	9.919576	11.20051	1.742467706	1.550326739	3.237782	3.211749
Mh-ERS1	1	1.00053	1.700204	1.50727706	1.010583055	0.533038	0.596976
	3	2.749966	1.254665	1.896673325	1.005513072	0.470818	0.640109
	5	2.277983	1.667125	1.808968104	1.079969058	0.427333	0.552795
	7	2.430599	2.212161	0.849811519	0.866569463	2.325932	1.573033
Mh-CTR1	1	1.001651	1.515929	0.652207146	1.002065273	0.79452	0.658383
	3	0.790037	0.342864	0.64049103	1.749972933	0.598439	2.395682
	5	0.907157	1.721104	0.372911705	0.906797443	1.074614	0.887891
	7	2.295902	0.708122	0.336321135	0.372821123	0.934528	0.802074
Mh-EIN2	1	1.003444	0.925949	1.571656432	1.00233122	1.407618	1.102971
	3	0.482512	0.320788	1.008969649	7.597127108	1.337639	1.95981
	5	1.592842	1.292483	1.239279544	4.50033996	2.51945	3.956159
	7	1.550534	1.256582	0.276311274	0.909127604	1.981878	8.2729
Mh-EIL1	1	1.001471	2.028234	1.930176729	1.000189835	1.035042	0.482167
	3	1.271945	1.511296	0.682891255	0.583902975	0.574408	0.623422
	5	2.160747	2.251	0.177106085	0.656476611	0.473263	3.284524
	7	2.616774	2.010442	1.610103872	0.297243893	0.520406	0.303224

Table S6. Primers used in qRT-PCR for genes related to ethylene biosynthesis and signal transduction.

Gene (GenBank Accession Number)	Primer Name	Sequence (5′→3′)
Mh-ACS1	BAS 5RT	ATGGACAGAGAGAGACGCTGAGAACC
(AF056162)	BAS 3RT	GAGGCTGTAGGCGATGTGAATCAG
Mh-ACO1	MAO1 5RT	ATGGCGATTCCGGTCATCGATTTCT
(AF004839)	MAO1 3RT	GAATCCCATGGTTCACCAGCTG
Mh-ACO2	MAO2 5RT	CAGCTCGAGGTAAATCACCAATGGC
(U80233)	MAO2 3RT	CCTCGGATAGACCTCCTTCTTCTC
Mh-ERS1	BR 5 RT	GTTGCCAGGGCAGATTACTTGAGG
(AF113747)	BR 3RT	GAATCCTCGACTTGCTACATTCCG
Mh-CTR1	De 5RT	AACAGTGCTTCCGGATCGCGTCTCTC
(JF430422)	De 3RT	CTTCCACATCAGTACATAGAGTCCAT
Mh-EIN2	BIN 5RT	CCAGCAACAATACACGGGTACCAG
(KX588216)	BIN 3RT	CGAGTTCAGTCCAGCCTGCCTATG
Mh-EIL1	177-5	CCCGGTTCTCTCTACGTCATCAAC
(KX588217)	177-3	GCACTTCCTAGAGTCAAGCTCTTG
Actin	BACT5	TAGCGACGTACCACAGGTAT
(AF246288)	BACT3	GTAAGCAAGCTTCTCCTTGAT
